# Binding of Dickkopf-3 to CXCR7 Enhances Vascular Progenitor Cell Migration and Degradable Graft Regeneration

**DOI:** 10.1161/CIRCRESAHA.118.312945

**Published:** 2018-06-06

**Authors:** Shirin Issa Bhaloo, Yifan Wu, Alexandra Le Bras, Baoqi Yu, Wenduo Gu, Yao Xie, Jiacheng Deng, Zhihong Wang, Zhongyi Zhang, Deling Kong, Yanhua Hu, Aijuan Qu, Qiang Zhao, Qingbo Xu

**Affiliations:** 1 From the School of Cardiovascular Medicine and Sciences, King’s College London British Heart Foundation Centre, United Kingdom (S.I.B., A.L.B., W.G., Y.X., J.D., Z.Z., Y.H., Q.X.); 2 State Key Laboratory of Medicinal Chemical Biology, Key Laboratory of Bioactive Materials, Ministry of Education, College of Life Sciences, Nankai University, Tianjin, China (Y.W., Z.W., D.K., Q.Z.); 3 Department of Physiology and Pathophysiology, Capital Medical University, Beijing, China (B.Y., A.Q.).

**Keywords:** animals, mice, rats, stem cells, tissue engineering

## Abstract

**Rationale:**

Vascular progenitor cells play key roles in physiological and pathological vascular remodeling—a process that is crucial for the regeneration of acellular biodegradable scaffolds engineered as vital strategies against the limited availability of healthy autologous vessels for bypass grafting. Therefore, understanding the mechanisms driving vascular progenitor cells recruitment and differentiation could help the development of new strategies to improve tissue-engineered vessel grafts and design drug-targeted therapy for vessel regeneration.

**Objective:**

In this study, we sought to investigate the role of Dkk3 (dickkopf-3), recently identified as a cytokine promotor of endothelial repair and smooth muscle cell differentiation, on vascular progenitor cells cell migration and vascular regeneration and to identify its functional receptor that remains unknown.

**Methods and Results:**

Vascular stem/progenitor cells were isolated from murine aortic adventitia and selected for the Sca-1 (stem cell antigen-1) marker. Dkk3 induced the chemotaxis of Sca-1+ cells in vitro in transwell and wound healing assays and ex vivo in the aortic ring assay. Functional studies to identify Dkk3 receptor revealed that overexpression or knockdown of chemokine receptor CXCR7 (C-X-C chemokine receptor type 7) in Sca-1+ cells resulted in alterations in cell migration. Coimmunoprecipitation experiments using Sca-1+ cell extracts treated with Dkk3 showed the physical interaction between DKK3 and CXCR7, and specific saturation binding assays identified a high-affinity Dkk3-CXCR7 binding with a dissociation constant of 14.14 nmol/L. Binding of CXCR7 by Dkk3 triggered the subsequent activation of ERK1/2 (extracellular signal-regulated kinases 1/2)-, PI3K (phosphatidylinositol 3-kinase)/AKT (protein kinase B)-, Rac1 (Ras-related C3 botulinum toxin substrate 1)-, and RhoA (Ras homolog gene family, member A)-signaling pathways involved in Sca-1+ cell migration. Tissue-engineered vessel grafts were fabricated with or without Dkk3 and implanted to replace the rat abdominal aorta. Dkk3-loaded tissue-engineered vessel grafts showed efficient endothelization and recruitment of vascular progenitor cells, which had acquired characteristics of mature smooth muscle cells. CXCR7 blocking using specific antibodies in this vessel graft model hampered stem/progenitor cell recruitment into the vessel wall, thus compromising vascular remodeling.

**Conclusions:**

We provide a novel and solid evidence that CXCR7 serves as Dkk3 receptor, which mediates Dkk3-induced vascular progenitor migration in vitro and in tissue-engineered vessels, hence harnessing patent grafts resembling native blood vessels.

## Abstract 2

Supplemental Digital Content is available in the text.

**Meet the First Author, see p 398**

Coronary and peripheral vascular bypass surgeries are one of the most effective treatments for cardiovascular diseases, in which autografts constitute the standard clinical approach for blood vessel replacement.^1^ However, because of disease or previous harvesting, autologous vessels have limited availability. As an alternative, tissue-engineered vessel grafts (TEVGs) composed of synthetic or biodegradable polymers have been developed.^2,3^ Although with apparent potential, their clinical application has been challenged by unsuccessful long-term patency because of intimal hyperplasia and thrombosis. The design of patent vascular grafts, capable of inducing vascular remodeling, adequately integrating into the native blood vessel, and displaying the required mechanical strength to withstand the blood pressure, greatly relies on host cell recruitment, particularly of vascular progenitor cells (VPCs).^4,5^

Accumulating evidence has shown that stem/progenitor cells play an active role in vascular remodeling.^6–9^ Among these cells, it has been demonstrated that vessel wall resident VPCs are able to migrate, proliferate, and differentiate into different cell lineages, including endothelial and smooth muscle cells (SMCs).^10–12^ Our group has identified a population of multipotent and lineage committed progenitor cells, for example, Sca-1 (stem cell antigen-1)+, c-kit+, CD (cluster of differentiation) 34+, and Flk1+ cells, resident in the aortic adventitia of ApoE (apolipoprotein E)-deficient mice.^13^ In experimental vein graft and wire-induced arterial injury mouse models, the adventitia-derived Sca-1+ progenitor cells transferred to the perivascular side of the vessel were able to migrate to the neointima and to differentiate into SMCs, with contribution to vascular remodeling and atherosclerotic lesion enhancement.^13,14^ More recently, a study demonstrated that a subpopulation of Sca-1+ cells originates from differentiated medial SMCs and that these cells not only exhibit a multipotent phenotype but can also expand and contribute to adventitial remodeling after vascular injury.^15^ However, little is known about the molecular mechanisms of VPC recruitment in vivo.

Dkk3 (dickkopf-3) is a secreted glycoprotein, highly expressed in endothelial cells (ECs), SMCs, and platelets.^16–19^ Functionally, Dkk3 was found to play important roles in cardiovascular biology. Previous reports had already demonstrated that Dkk3 can protect against cardiac hypertrophy,^20,21^ and our group recently showed that Dkk3 is an atheroprotective cytokine. Indeed, in a human population-based prospective study, Dkk3 serum level was inversely correlated with carotid artery intimal thickening.^19^ These findings, together with the potential of Dkk3 to induce SMC differentiation of stem cells,^16,17^ led us to investigate its effect on VPCs and vascular regeneration, which remains unknown. Mechanistically, a great controversy surrounds the identification of Dkk3 receptor. Some studies have shown that, contrary to Dkk1, Dkk2, and Dkk4, Dkk3 is not able to bind to Wnt pathway components Kremen1, Kremen2, LRP (low-density lipoprotein receptor–related protein) 5, and LRP6.^22–26^ On the contrary, contradictory reports have indicated that Dkk3 could interact with these receptors.^16,25,27^ We hypothesized that Dkk3 could act as a chemokine for VPCs possibly via binding to a chemokine receptor. In this work, we identify CXCR7 (C-X-C chemokine receptor type 7) as the functional chemokine receptor for Dkk3 and elucidate the downstream migration-related pathways ERK1/2 (extracellular signal-regulated kinases 1/2), PI3K (phosphatidylinositol 3-kinase)/AKT (protein kinase B), and Rho GTPases. We also designed Dkk3-loaded TEVGs and showed that they exhibit long-term patency with controlled recruitment and differentiation of VPCs, thus harnessing neoarteries resembling native blood vessels. CXCR7 blocking in this model inhibits VPC recruitment and compromises graft remodeling.

## Methods

The data that support the findings of this study are available from the corresponding author on reasonable request.

Details of materials and experimental procedures are available in the Online Data Supplement.

### Sca-1+ Adventitial Progenitor Cell Isolation and Dkk3 Binding

Mouse VPCs were isolated from the outgrowth of aortic adventitial tissues as described previously.^13^ Sca-1+ VPCs were treated with 25 ng/mL of Dkk3 for 3 hours. The lysate was precleared by using the control agarose resin column. The eluted immune complexes and input samples were separated on a 4% to 12% Bis-Tris gel, and the immunoblot was probed with Dkk3 antibody. His-tag pull-down assay was performed according to the instructions provided in MagneHis Protein Purification System (Promega). Receptor affinity binding assay was done as described previously.^22^

## Results

### Dkk3 Induces Migration of Resident Sca-1+ VPCs In Vitro and Ex Vivo

**Figure 1.**
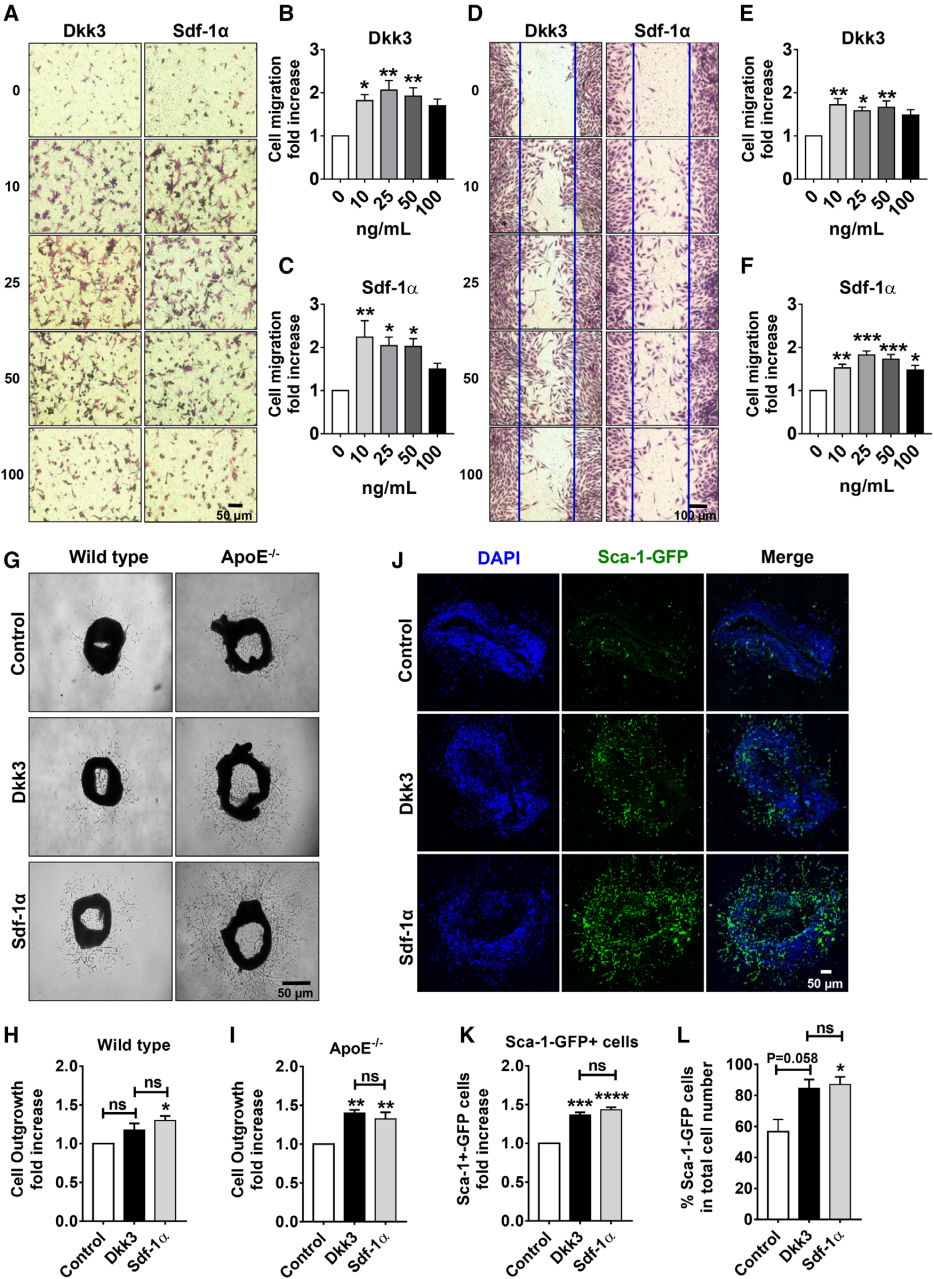
**Dkk3 (dickkopf-3) induces migration of resident Sca-1 (stem cell antigen-1)+ vascular progenitor cells in vitro and ex vivo. A**, Representative images of migrated Sca-1+ cells induced by increasing concentration of either Dkk3 or Sdf-1α (stromal cell-derived factor 1α; 0, 10, 25, 50, and 100 ng/mL), in a transwell migration assay performed during 12 h (×20 magnification). **B** and **C**, Quantification of the transwell assay with Dkk3 and Sdf-1α stimulation, respectively (n=5). **D**, Representative images of migrated Sca-1+ cells, 16 h after the initiation of wound healing assay, at increasing concentration of either Dkk3 or Sdf-1α (0, 10, 25, 50, and 100 ng/mL). Blue solid lines denote the margins of the wound (×20 magnification). **E** and **F**, Quantification of cells that migrated to close the wounded area in response to Dkk3 and Sdf-1α, respectively (n=5). Both migration assays showed that Dkk3 induces Sca-1+ cell migration and that the chemotactic effect of Dkk3 is similar to the observed with Sdf-1α stimulation. **G**, Representative images of cell outgrowth from aortic rings from wild-type and ApoE (apolipoprotein E)^−/−^ mice induced by either Dkk3 (25 ng/mL) or Sdf-1α (25 ng/mL) treatment. **H** and **I**, Quantitative analysis of cell outgrowth from wild-type and ApoE^−/−^ mice aortic rings, respectively (n=4). Dkk3 induces cell outgrowth from ApoE^−/−^ aortic rings, whereas Sdf-1α induces cell outgrowth from both wild-type and ApoE^−/−^ explants. **J**, Representative immunofluorescence images of aortic rings from transgenic Sca-1-GFP (green fluorescent protein) ApoE^−/−^ mice treated with either Dkk3 (25 ng/mL) or Sdf-1α (25 ng/mL). DAPI (4,6-diamidino-2-phenylindole) was included to counterstain the nuclei (×20 magnification). **K**, Quantification of the number of Sca-1+-GFP cells that outgrew from the aortic rings (n=3). **L**, Quantification of the percentage of Sca-1+ GFP cells against the total number of cells present in the outgrowth (n=3). Dkk3 promotes Sca-1+ cell outgrowth from ApoE^−/−^ aortic rings in a similar manner as Sdf-1α does. The data are expressed as the mean±SEM of 5 independent experiments for the in vitro migration assays (**A**–**F**) and of 3 to 4 independent experiments for the ex vivo migration assays (**G**–**L**). NS indicates nonsignificant. *P<0.05, **P<0.01, ***P<0.001, ****P<0.0001, compared with control group (0 ng/mL; 1-way ANOVA followed by Bonferroni post hoc test).

To investigate the chemotactic potential of Dkk3 in Sca-1+ cells, we first performed in vitro migration assays. Sdf-1α (stromal cell-derived factor 1α) chemokine was used as a positive control for cell migration in the experiments.^28,29^ Transwell and wound healing assays revealed that Dkk3 markedly induced the migration of Sca-1+ cells over a range of concentrations of 0 to 100 ng/mL (Figure 1A, 1B, 1D, and 1E), indicating that Dkk3 possesses chemokine-like properties comparable with Sdf-1α (Figure 1A, 1C, 1D, and 1F). To rule out a potential contribution of Dkk3-induced Sca-1+ cell proliferation in our observations, we performed bromodeoxyuridine incorporation cell proliferation assay at 16, 24, and 48 hours of treatment. No significant differences were observed in the cell proliferation rate of the cells between control and treatment groups, when the dose of 25 ng/mL of Dkk3 was used (Online Figure IA). Dkk3 treatment at higher concentration (50 ng/mL) promoted cell proliferation only after 48 hours of treatment. The effect of Dkk3 on cell death by apoptosis and necrosis was also determined. During the 24 hours of treatment (25 ng/mL), no significant difference in cell death rate was found between control and Dkk3 groups (Online Figure IB). We thus concluded that Dkk3 induces the chemotaxis of Sca-1+ cells in vitro. To further assess the chemotactic effect of Dkk3 in a setting that better mimics the in vivo 3-dimensional conditions and to study the effect of Dkk3 on endogenous resident VPCs of the vessel wall, the aortic ring assay was performed with aortas of wild-type and ApoE^−/^^−^ mice. In wild-type explants, although Dkk3 induced cell outgrowth, no difference with statistical significance was observed between control and Dkk3-treated group (Figure 1G and 1H). However, in ApoE^−/−^ explants, Dkk3 remarkably promoted cell outgrowth from the aortic rings, analogously to Sdf-1α (Figure 1G and 1I). The explanation could be that vascular cells derived from the native atherosclerosis model ApoE^−/−^ mice are potentially more responsive or sensitive to stimuli than the cells derived from wild-type mice. We have shown in a previous study that, compared with wild-type cells, ApoE^−/−^ Sca-1+ cells exhibit an altered expression of genes implicated in cell migration, including matrix metallopeptidases and integrins, and possess an intrinsically enhanced migratory potential.^30^ Because cell outgrowth from the aortic rings comprises different cell types, we next sought to examine the contribution of Sca-1+ progenitor cells in Dkk3-induced outgrowth. Aortic rings from transgenic Sca-1-GFP (green fluorescence protein) ApoE^−/−^ mice were isolated and cultured because this mouse model allows rapid detection of Sca-1+ cells, through GFP expression. Dkk3 treatment triggered an increase in Sca-1+ GFP+ cell outgrowth compared with control (Figure 1J and 1K). Moreover, the percentage of Sca-1+ GFP+ cells in the total number of outgrown cells was also higher in Dkk3 treatment group than in control group (Figure 1J and 1L). These results further confirmed the chemotactic role of Dkk3 in resident Sca-1+ VPCs with an efficiency comparable with Sdf-1α.

### CXCR7 Is Involved in Dkk3-Driven Migration of Sca-1+ Cells

**Figure 2.**
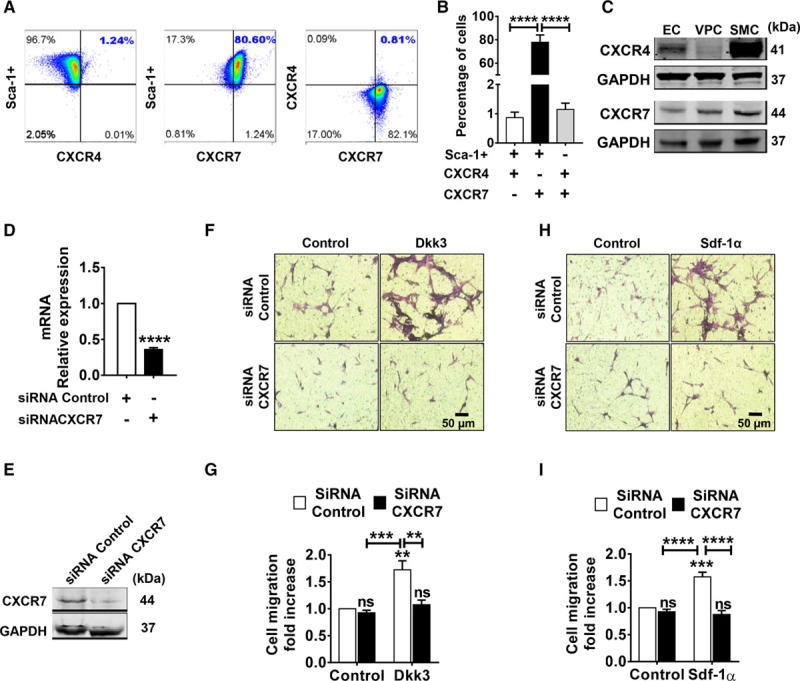
**CXCR7 is involved in Dkk3 (dickkopf-3)-driven migration of Sca-1 (stem cell antigen-1)+ cells. A**, Representative flow cytometry dot plots of CXCR4 (C-X-C chemokine receptor type 4) and CXCR7 (C-X-C chemokine receptor type 7) surface expression in Sca-1+ cells. **B**, Quantification of Sca-1+/CXCR4+, Sca-1+/CXCR7+, and Sca-1+/CXCR7+/CXCR4+ cells by flow cytometry analysis (n=3; 1-way ANOVA followed by Bonferroni post hoc test). CXCR7 is expressed in 78% (77.9±6.2%) of Sca-1+ cells, whereas CXCR4 is expressed in only 1% (0.9±0.2%) of the cells. **C**, Western blot analysis of CXCR4 and CXCR7 expression in murine endothelial cells (ECs), Sca-1+ cells (vascular progenitor cell [VPC]), and smooth muscle cells (SMC). CXCR7 is highly expressed in Sca-1+ cells and SMCs. CXCR4 expression in Sca-1+ cells is low in comparison with its expression in ECs and SMCs. **D**, Quantitative polymerase chain reaction analysis of CXCR7 expression in Sca-1+ cells transfected with siRNA targeting CXCR7. Expression levels were normalized against GAPDH (n=5; 2-tailed Student *t* test). **E**, Western blot analysis of CXCR7 knockdown in Sca-1+ cells transfected with CXCR7 siRNA. **F** and **H**, Representative images of transwell migration assay of Sca-1+ cells transfected with CXCR7 siRNA in response to Dkk3 (25 ng/mL) and Sdf-1α (stromal cell-derived factor 1α; 25 ng/mL) treatment, respectively. **G** and **I**, Quantitative analysis of the migrated cells in response to Dkk3 or Sdf-1α treatment (n=4; 2-way ANOVA followed by Bonferroni test). CXCR7 knockdown in Sca-1-VPCs supresses Dkk3-mediated migration, similarly to the observed with Sdf-1α treatment. The data are expressed as the mean±SEM of 3 to 5 independent experiments. NS indicates nonsignificant. **P<0.01, ***P<0.001, ****P<0.0001, compared with control group (0 ng/mL).

The similarity of action between Dkk3 and Sdf-1α prompted us to investigate whether the receptors of Sdf-1α, CXCR4 (C-X-C chemokine receptor type 4), and CXCR7 could participate in Dkk3-mediated migration of Sca-1+ cells. Flow cytometry analysis revealed that most of Sca-1+ cells expressed CXCR7 (77.9±6.2%), whereas few Sca-1+ cells displayed CXCR4 on their surface (0.9±0.2%; Figure 2A and 2B). Few cells were positive for both receptors (1.1±0.2%), and all the cells expressing CXCR4 also expressed CXCR7. Western blot analysis further confirmed the high expression of CXCR7 in Sca-1+ cells (Figure 2C). Noticeably, contrary to Sca-1+ cells, SMCs and ECs significantly expressed both CXCR4 and CXCR7. We speculate that CXCR7 may potentially be a vascular stem cell receptor, which is supported by previous studies that report a high expression of CXCR7 in progenitor cells.^31–33^ To assess the potential involvement of CXCR7 in Dkk3- and Sdf-1α-driven Sca-1+ cell migration, we performed transwell migration assays on progenitors after efficient knockdown of CXCR7 by siRNA targeting. Downregulation of CXCR7 was confirmed by quantitative polymerase chain reaction (Figure 2D) and Western blot (Figure 2E) analysis. CXCR7 downregulation induced strong decrease in Dkk3-driven Sca-1+ cell migration (Figure 2F and 2G), comparable with the decrease observed in Sdf-1α–driven cell migration (Figure 2H and 2I). Our findings proved that CXCR7 was involved in Sca-1+ cell migration induced by Dkk3. Because of the low expression of CXCR4, a different strategy was used to inhibit its functional activity. Migration assays were performed using CXCR4 inhibitor AMD3100, at 3 different concentrations: 10, 25, and 50 µmol/L (Online Figure IIA and IIB). Inhibition of CXCR4 had no effect on the migration rate of Sca-1+ cells in the presence of either Dkk3 or Sdf-1α. This suggested that CXCR4 receptor was not implicated in either Dkk3- or Sdf-1α–mediated migration of Sca-1+ cells.

### CXCR7 Is a High-Affinity Binding Receptor of Dkk3

**Figure 3.**
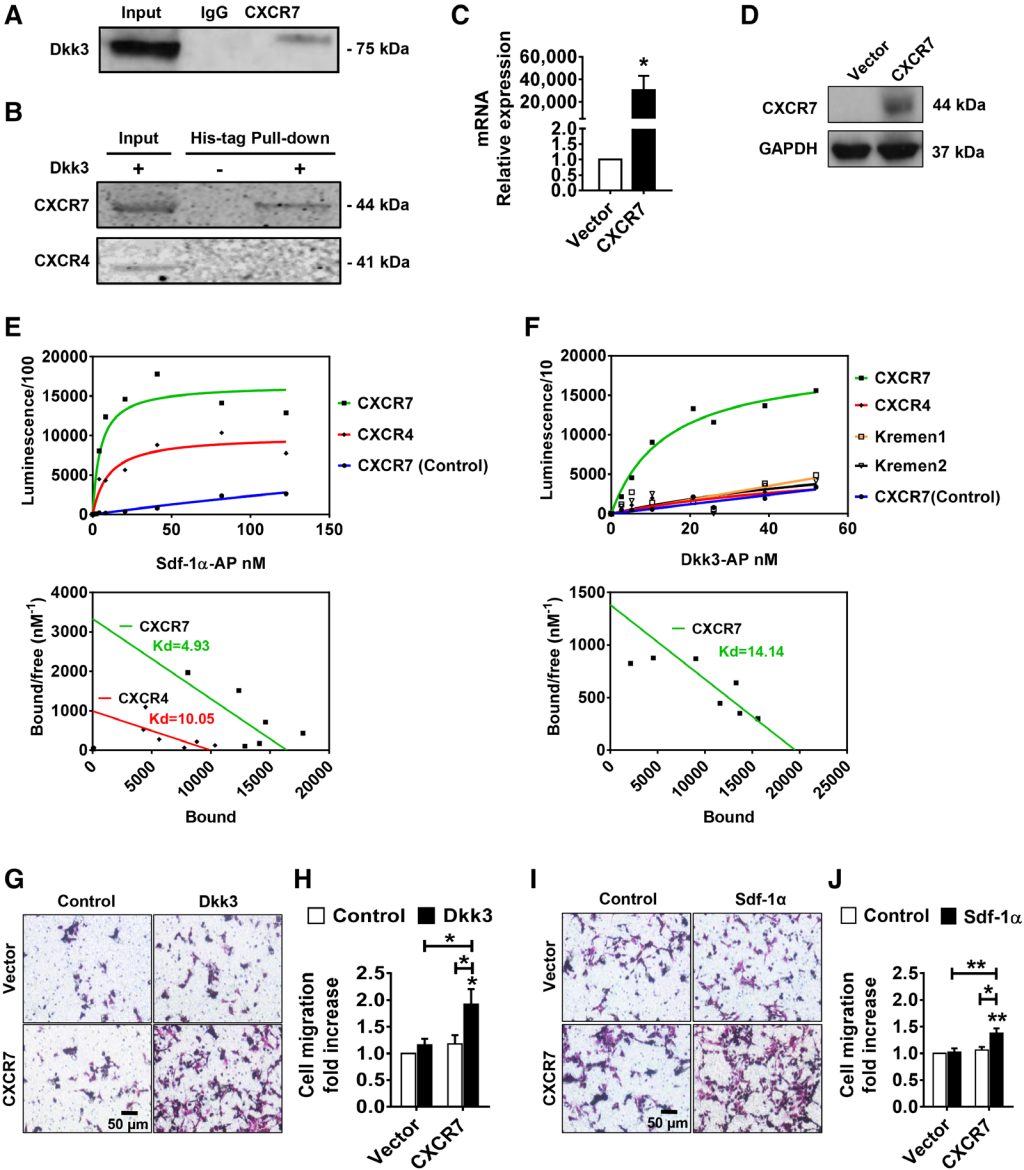
**CXCR7 is a high-affinity binding receptor of Dkk3 (dickkopf-3). A**, Coimmunoprecipitation of Dkk3 with CXCR7. Dkk3 coimmunoprecipitates with CXCR7 from Sca-1 (stem cell antigen-1)+ cells. **B**, Immunoblotting of the receptors pulled down from Dkk3-His tagged, using Nickel magnetic beads. Sca-1+ cells were treated with Dkk3-His tagged. CXCR7, but not CXCR4, is pulled down with Dkk3-His. **C**, Quantitative real-time quantitative polymerase chain reaction analysis of CXCR7 mRNA expression in HEK 293T cells transfected with CXCR7 expression plasmid. Expression levels were normalized against GAPDH (n=4; 2-tailed unpaired Student *t* test). **D**, Western blot analysis of CXCR7 overexpression in HEK 293T cells transfected with CXCR7 expression plasmid. **E** and **F**, Representative binding curves and respective Scatchard analysis of Sdf-1α (stromal cell-derived factor 1α)–alkaline phosphatase (AP) binding to CXCR4 or CXCR7 overexpressed in HEK 293T cells and of Dkk3-AP binding to CXCR7, CXCR4, Kremen1, or Kremen2 overexpressed in HEK 293T cells, respectively. The dissociation constants are represented for each receptor (n=3). Dkk3-AP does not bind to CXCR4, Kremen1, and Kremen2, but it does bind with high affinity to CXCR7, as represented by the characteristic hyperbolic binding curve. CXCR7 is also a high-affinity binding receptor of Sdf-1α, alongside its cognate receptor CXCR4. AP alone does not bind to CXCR7, as depicted in blue. **G** and **I**, Representative images of transwell migration assay of HEK 293T cells overexpressing CXCR7 in response to Dkk3 and Sdf-1α stimulation, respectively. **H** and **J**, Quantitative analysis of the transwell migration assays. Dkk3 induces migration of CXCR7-overexpressing HEK 293T cells, analogously to Sdf-1α. (n=5; 2-way ANOVA followed by Bonferroni post hoc test). The data are expressed as the mean±SEM of 3 to 5 independent experiments. *P<0.05, **P<0.01, compared with control group.

To verify the physical interaction between Dkk3 and CXCR7, we performed a coimmunoprecipitation assay. CXCR7 from lysates of Sca-1+ cells treated with Dkk3 was pulled down using anti-CXCR7 antibody and coprecipitated with Dkk3, as revealed by Western blot, with a corresponding band displayed in the input sample and not when an IgG control antibody was used (Figure 3A). We also conducted a reverse pull-down assay, in which Dkk3-His tagged was pulled down from Sca-1+ cell lysate and eluted with any bound CXCR7 (Figure 3B). Noticeably, contrarily to CXCR7, CXCR4 was not pulled down with Dkk3. To measure the affinity of the interaction between Dkk3 and CXCR7, saturation binding experiments were performed as described previously.^22,23^ The measurement of Sdf-1α–binding affinity to CXCR4 and CXCR7 was used to validate the design of the experiment. Dkk3 and Sdf-1α were conjugated with alkaline phosphatase (AP), and the efficiency of AP conjugation was confirmed by determining the AP activity after serial dilutions (Online Figure IIIA and IIIB). Next, CXCR7 and CXCR4 receptors (HA tagged) were overexpressed in HEK (human embryonic kidney) 293T cells (Figure 3C and 3D; Online Figure IIC and IID), and the corresponding cell lysates were loaded onto the wells of high-affinity–binding ELISA plates previously coated with anti-HA (hemagglutinin) antibody. Sdf-1α-AP bound with high affinity to CXCR4 and CXCR7 as characteristic hyperbolic curves were obtained for each receptor (Figure 3E). The dissociation constant for CXCR4 (10.05 nmol/L) was comparable with that calculated for CXCR7 (4.93 nmol/L), indicating comparable strength of affinity for Sdf-1α. Dkk3-AP also bound with high affinity to CXCR7, which was represented by the typical hyperbolic binding curve and a dissociation constant of 14.14 nmol/L (Figure 3F). The binding curve observed for CXCR4 was also indicative of absence of Dkk3 binding. To rule out the possibility that AP itself could bind to CXCR7, a serial dilution of AP alone was performed. The results showed only a residual binding (Figure 3E and 3F). Because Kremen1 and Kremen2 were identified as functional receptors for members of the Dkk family,^22^ we also assessed their binding affinity to Dkk3. Sca-1+ VPCs express Kremen1 but display a low expression of Kremen2 (Online Figure IVA and IVB). In addition, Kremen1 was neither pulled down with Dkk3-His-tagged (Online Figure IVC) nor it coimmunoprecipitated with CXCR7 (Online Figure IVD), in Sca-1+ VPCs. In the saturation binding assay, ambiguous binding curves were obtained for Kremen1 and Kremen2, suggesting that Dkk3 could not bind to these receptors (Figure 3E). . Kremen1 downregulation by SiRNA transfection also did not affect significant Dkk3-induced migration of Sca-1+ cells (Online Figure IVE through IVG). Finally, Dkk3 also increased the migration rate of CXCR7-overexpressing HEK cells in comparison with the nontreated control group (Figure 3G and 3H), whereas overexpression of Kremen1 and Kremen2 did not modify Dkk3-mediated HEK cell migration (Online Figure IVH through IVM). Treatment with Sdf-1α also resulted in an increase of the migration rate of CXCR7-overexpressing cells (Figure 3I and 3J). Not surprisingly, Sdf-1α induced the migration of cells overexpressing CXCR4, when compared with nontreated control group (Online Figure IIG and IIH). However, Dkk3 treatment had no effect on the migration rate of CXCR4-overexpressing cells (Online Figure IIE and IIF). We thus concluded that Dkk3 not only binds with high affinity to CXCR7 but also that CXCR7 is a functional receptor for Dkk3.

### ERK1/2 and PI3K/AKT Signaling Pathways Act Downstream of Sca-1+ Cell Migration

**Figure 4.**
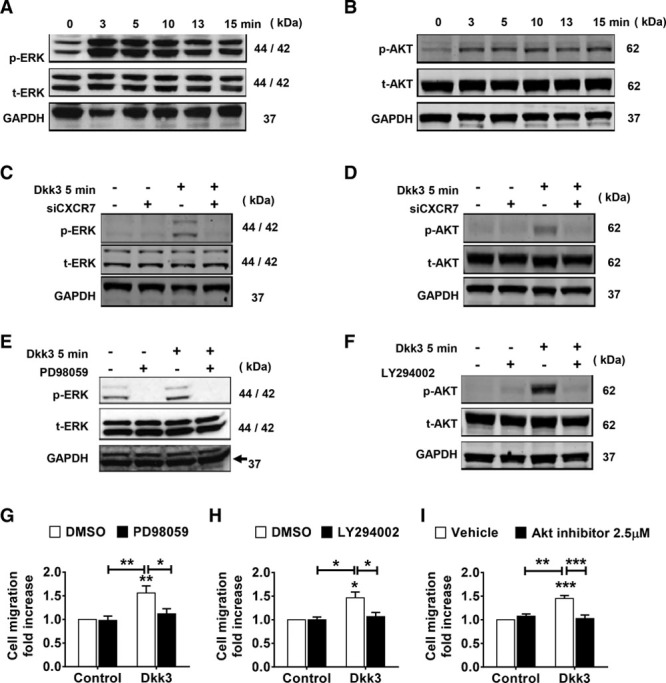
**ERK1/2 (extracellular signal-regulated kinases 1/2) and PI3K (phosphatidylinositol 3-kinase)/AKT signaling pathways act downstream of Sca-1 (stem cell antigen-1)+ cell migration mediated by Dkk3 (dickkopf-3) binding to CXCR7. A** and **B**, Western blot analysis of phosphorylated and total ERK1/2 and AKT proteins in Sca-1+ cells stimulated with Dkk3 at the indicated time points. ERK1/2 and AKT phosphorylation increases in response to Dkk3. **C** and **D**, Western blot analysis of Dkk3-induced ERK1/2 and AKT activation in Sca-1+ cells after CXCR7 knockdown by siRNA transfection. Downregulation of CXCR7 abolishes Dkk3-triggered phosphorylation of ERK1/2 and AKT. **E** and **F**, Western blot analysis of ERK1/2 and AKT activation in Sca-1+ cells stimulated with Dkk3 for 5 min and pretreated with PD98059 and LY294002, respectively. ERK1/2 and AKT activation is supressed on treatment with the inhibitors. **G**–**I**, Quantification of Dkk3-driven Sca-1+ cell migration on treatment with PD98059 (10 µmol/L; n=5), LY294002 (10 µmol/L; n=3), and AKT inhibitor (2.5 µmol/L; n=4), respectively. Dkk3-driven cell migration is abrogated in response to all inhibitors. Western blot images are representative of 3 independent experiments. The data are expressed as the mean±SEM of 3 or 5 independent experiments. *P<0.05, **P<0.01, ***P<0.001, compared with control group (0 ng/mL; 2-way ANOVA followed by Bonferroni post hoc test).

MAPK (mitogen-activated protein kinase) kinases (such as ERK1/2) and PI3K/AKT pathways are classical signaling cascades involved in cell migration triggered by the activation of chemokine receptors.^34,35^ Having established the chemotactic role of Dkk3 and its effective binding to chemokine receptor CXCR7, we next sought to investigate whether these pathways were involved in Sca-1+ cell migration induced by Dkk3 binding to CXCR7. Changes in the phosphorylation level of ERK1/2 and AKT were examined by Western blot after 0 to 15 minutes of Dkk3 stimulation. Dkk3 treatment induced ERK1/2 and AKT phosphorylation (Figure 4A and 4B). Stimulation with Sdf-1α also increased ERK1/2 and AKT phosphorylation (Online Figure VA and VC). Sdf-1α–mediated ERK1/2 and AKT phosphorylation and consequent induction of cell migration have been shown before in previous studies.^36–38^ We provide solid evidence of the implication of ERK1/2 and PI3K/AKT pathways in the mechanisms of cell migration promoted by Dkk3. Treatment with PD98059 and LY294002 inhibited, respectively, ERK1/2 and AKT phosphorylation induced by Dkk3 (Figure 4E and 4F) and Dkk3-induced cell migration (Figure 4G and 4H). Specific AKT inhibitor (AKT inhibitor X) also decreased Dkk3-driven migration of Sca-1+ cells (Figure 4I). Furthermore, knockdown of Akt and Erk pathways was performed by SiRNA transfection (Online Figure 6). Altogether, our data showed that ERK1/2- and PI3K/AKT-signaling pathways were required for Sca-1+ cell migration induced by Dkk3. Interestingly, Sdf-1α–mediated migration of Sca-1+ cells shared the same signaling mechanisms (Online Figure VA through VE). Furthermore, CXCR7 siRNA-mediated downregulation in Sca-1+ cells led to a decrease in Dkk3-driven ERK1/2 and AKT phosphorylation (Figure 4C and 4D), which showed that CXCR7 activation by Dkk3 acts upstream these signaling pathways.

### Rac1 and RhoA GTPase Activation Is Required

**Figure 5.**
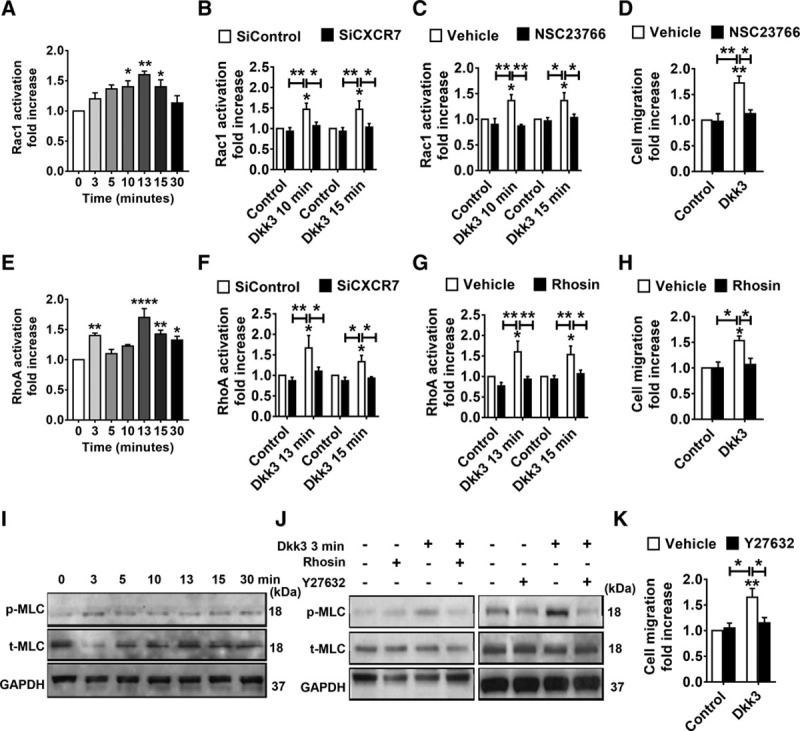
**Rac1 (Ras-related C3 botulinum toxin substrate 1) and RhoA (Ras homolog gene family, member A) GTPase activation is required for Sca-1 (stem cell antigen-1)+ cell migration induced by Dkk3 (dickkopf-3) binding to CXCR7. A** and **E**, Quantification of Rac1-GTP and RhoA-GTP in Sca-1+ cells treated with Dkk3 at the time points indicated. Dkk3 induces Rac1 and RhoA activation. **B** and **F**, Quantification of Dkk3-triggered activation of Rac1 and RhoA after CXCR7 knockdown by siRNA transfection. Downregulation of CXCR7 abolishes Dkk3-triggered activation of Rac1 and RhoA (n=3). **C**, Quantification of Dkk3-induced Rac1 activation on treatment with NSC23766 (10 µmol/L; n=3). Dkk3-induced increase of Rac1-GTP level at 10 and 15 min is repressed by the inhibitor NSC23766. **D**, Quantification of Dkk3-driven Sca-1+ cell migration on NSC23766 treatment. NSC23766 inhibitor abolishes Sca-1+ cell migration promoted by Dkk3 treatment (n=4). **G**, Quantification of Dkk3-induced RhoA activation on Rhosin (10 µmol/L; n=3) treatment. Dkk3-induced increase of RhoA-GTP level is repressed by the inhibitor Rhosin, at the time points indicated. **H**, Quantification of Dkk3-driven Sca-1+ cell migration on Rhosin treatment (n=3). Rhosin abolishes Sca-1+ cell migration promoted by Dkk3 treatment. **I**, Western blot analysis of MLC (myosin light chain) phosphorylation in response to Dkk3 stimulation. MLC phosphorylation is induced by Dkk3 at the time points indicated. **J**, Western blot analysis of Dkk3-driven MLC phosphorylation on pretreatment with Rhosin or Y27632, at the time point indicated. Dkk3-induced MLC phosphorylation is repressed in response to the inhibitors. **K**, Quantification of Dkk3-driven Sca-1+ cell migration on Y27632 treatment. Sca-1+ cell migration promoted by Dkk3 treatment is inhibited by Y27632 (5 µmol/L; n=4) treatment. The Western blot images are representative of 3 independent experiments. The data are expressed as the mean±SEM of 3 to 4 independent experiments. *P<0.05, **P<0.01, ****P<0.0001, compared with control group (0 ng/mL; 1-way ANOVA for **A** and **E** and 2-way ANOVA for **B**, **C**, **D**, **F**, **G**, **H**, and **K**, followed by Bonferroni post hoc test).

Rho GTPases are well known for their role in cytoskeleton rearrangement and cell migration. Quantification of the active GTP-bound Rac1 (Ras-related C3 botulinum toxin substrate 1) and RhoA (Ras homolog gene family, member A) forms in Sca-1+ cells was assessed by performing G-LISA activation assay at early time points of Dkk3 stimulation (0–30 minutes) and showed that Dkk3 triggered the activation of Rac1 and RhoA (Figure 5A and 5E). Transwell migration assays were performed in the presence of Rac1 activation inhibitor NSC23766. NSC23766 efficiently inhibited Rac1 activation (Figure 5C) and resulted in a decrease in the migration rate of Sca-1+ cells induced by Dkk3 (Figure 5D). Treatment of Sca-1+ cells with RhoA inhibitor Rhosin blocked Dkk3-induced RhoA activation (Figure 5G) and abolished Dkk3-mediated Sca-1+ cell migration (Figure 5H). SiRNA-mediated knockdown of CXCR7 in the cells abolished the Dkk3-triggered activation of Rac1 and RhoA (Figure 5B and 5F), which reveals that Rho GTPase activation induced by Dkk3 requires Dkk3 binding to CXCR7. The phosphorylation level of MLC (myosin light chain)—a downstream effector of RhoA—in response to Dkk3 stimulation was also assessed. In the presence of Dkk3, MLC phosphorylation at early time points was increased (Figure 5I). ROCKs (Rho-associated kinases) are activated by RhoA and can induce phosphorylation of MLC. Therefore, we investigated whether ROCK inhibitor Y27632 could repress MLC phosphorylation. Y27632 not only reduced Dkk3-triggered phosphorylation of MLC (Figure 5J) but also decreased the migratory ability of Sca-1+ cells stimulated by Dkk3 (Figure 5K). Finally, because RhoA can also directly phosphorylate MLC, we examined whether Rhosin could affect MLC activation. Inhibition of RhoA with Rhosin reduced the Dkk3-triggered phosphorylation of MLC (Figure 5J). Collectively, these results showed that Rac1 and RhoA GTPases are involved in Sca-1+ cell migration mediated by CXCR7 activation induced by Dkk3 binding. Finally, to investigate whether Rac1 and ERK1/2 signaling pathways were interacting with each other, we measured the Dkk3-driven phosphorylation level of ERK1/2 in the presence of Rac1 inhibitor and the Dkk3-triggered activation of Rac1 on treatment with ERK inhibitor. Dkk3-induced phosphorylation of ERK1/2 was supressed in response to NSC23766 treatment (Online Figure VIIA). Surprisingly, Rac1 activation triggered by Dkk3 was also repressed on treatment with PD98059 (Online Figure VIIB). These results indicated a possible feedback mechanism between ERK1/2 and Rac1. In contrast, Rac1 and RhoA activation stimulated by Dkk3 was not affected on treatment with PI3K/AKT inhibitor LY294002 (Online Figure VIIC and VIID). Interestingly, treatment with Sdf-1α also promoted the activation of Rac1 and RhoA and phosphorylation of MLC (Online Figure VF through VH).

Because Dkk proteins have been associated with the Wnt-signaling pathway,^39,40^ we assessed whether this pathway was implicated in Dkk3-driven cell migration. Dkk3 stimulation had no effect in the expression of β-catenin, Axin-2, and Tcf1 (Online Figure VIIIA through VIIID). Furthermore, 24 hours of Dkk3 stimulation did not activate the transcription of Wnt target genes, given that the luciferase activity did not modify in the presence of Dkk3 (Online Figure VIIIE). Treatment of Sca-1+ cells with FH535—a specific inhibitor of the canonical Wnt/β-Catenin signaling transduction pathway—resulted in an increase of cell migration, comparable with the increase obtained when the cells were treated with Dkk3 (Online Figure VIIIF). However, when cells were treated with Dkk3 combined with FH535, no cumulative effect and difference were found in the cell migration rate between this group and the group treated only with Dkk3. Inhibition of Dvl-PDZ (dishevelled protein—a critical component of both canonical and noncanonical Wnt pathways—with peptide Pen-N3), also did not affect Dkk3-triggered migration of Sca-1+ cells (Online Figure VIIIG) because no difference was found between Dkk3-treated group and the group treated with Dkk3 combined with peptide Pen-N3. Therefore, we concluded that the Wnt-signaling pathway is not involved in Sca-1+ cell migration induced by Dkk3.

### Characterization and Patency of Dkk3-Loaded TEVG in a Rat Model

To evaluate the role of Dkk3 in cell migration in vivo, TEVGs were evaluated by rat abdominal aorta replacement model (Online Figure IXA, left, top). Dkk3-loaded TEVGs (2.0 mm in diameter and 500 µm in wall thickness) were fabricated by coelectrospinning technique, then implanted into the abdominal artery of rats and examined at different time points. Scanning electronic microscopy showed homogeneous fibrous structure of the grafts (Online Figure X), consisting of polycacoprolactone microfibers (≈6 μm) and collagen nanofibers (≈600 nm). polycaprolactone microfibers provided good mechanical support and macroporous structure, whereas collagen nanofibers introduced a biocompatibility and acted as carriers for Dkk3. With the degradation of the collagen fibers, Dkk3 was released from the grafts in a sustained manner, and no burst release was observed during the detection period (Online Figure XI). Unloaded grafts were used as controls for comparison to determine the effect of loaded Dkk3. Ultrasound analysis at 2 weeks, 1 month, and 3 months (Online Figure IXA, right) indicated that most of the implanted grafts were patent and presented sustained blood flow, without aneurysm, bleeding, or occlusion (Online Figure IXC). Some capillaries could be observed from the adventitia of the implanted grafts, which reflects their integration with the surrounding tissues (Online Figure IXA, left, bottom). Micro-CT revealed that no detectable calcification occurred in both groups 3 months post-implantation (Online Figure IXB).

### Delivery of Dkk3 in TEVGs Enhances Vascular Regeneration

**Figure 6.**
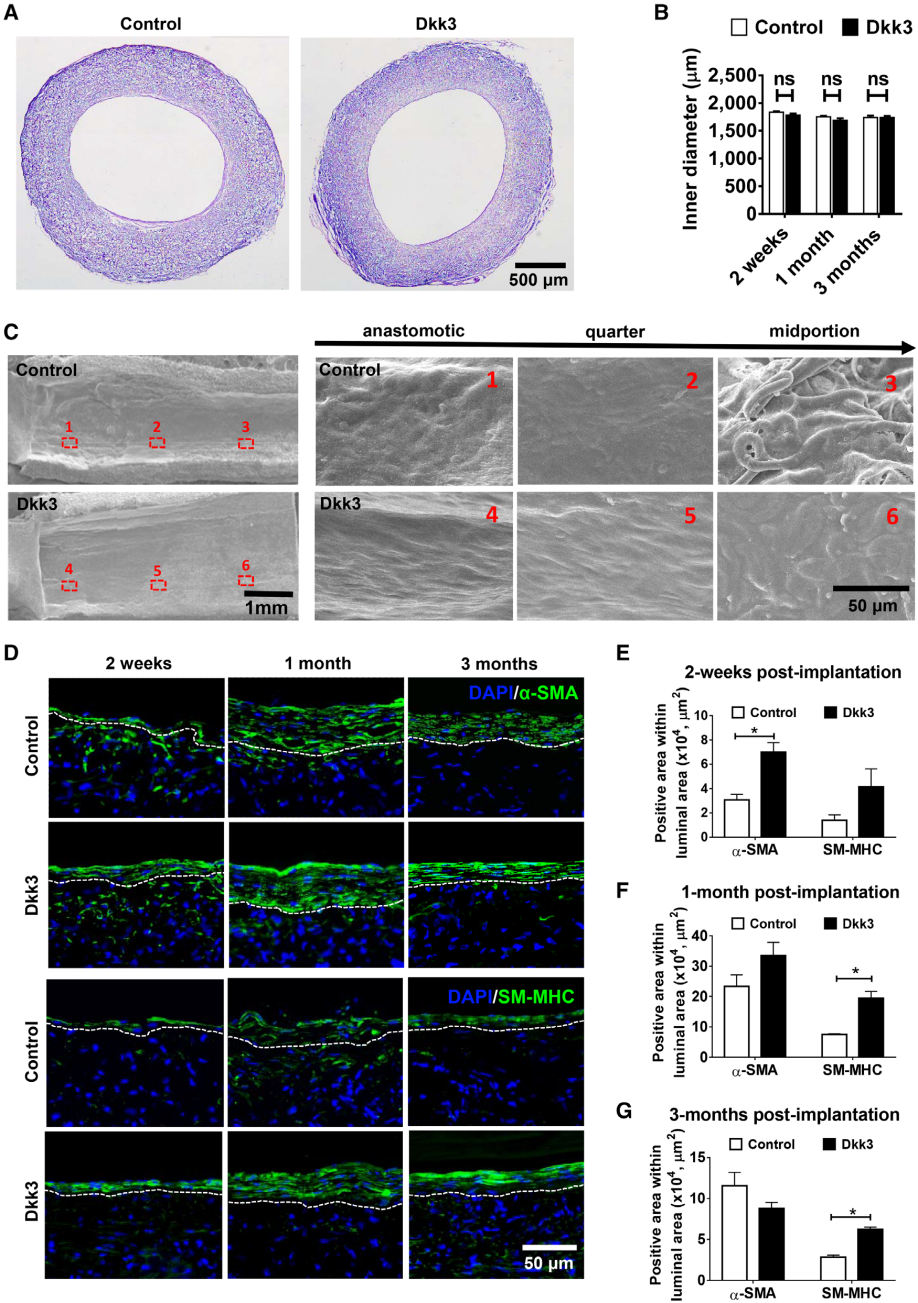
**Delivery of Dkk3 (dickkopf-3) in tissue-engineered vascular grafts (TEVGs) enhances vascular regeneration.** Dkk3-loaded TEVGs were implanted into rats to replace the abdominal artery and then harvested 2 wk, 1 mo, and 3 mo post-implantation. **A**, H&E staining on cross sections of control and Dkk3-loaded TEVGs 3 mo post-implantation. Both grafts are fully cellularized, and no evident intimal hyperplasia is observed. **B**, Quantification of the inner diameter of the TEVGs at 2 wk, 1 mo, and 3 mo. The inner diameters of the grafts remain unchanged ≤3 mo post-implantation. **C**, Representative images of the luminal surface of the explanted TEVGs, 3 mo post-implantation, obtained by scanning electronic microscopy. Three locations were selected continuously from the anastomotic sites to the middle of the grafts, and the respective magnified images are shown. The surface of Dkk3-TEVGs throughout the graft is more continuous and homogeneous in comparison with the control group. **D**, Representative immunofluorescence images of control- and Dkk3-TEVGs showing SMC recruitment by staining the sections with anti–α-SMA (α-smooth muscle actin) and anti-SM-MHC (smooth muscle myosin heavy chain) antibodies. DAPI was used to counterstain the nuclei. **E**–**G**, Quantification of smooth muscle regeneration by determination of the total area of α-SMA and of SM-MHC–positive cells in the luminal region (delineated by the white dashed lines) at different time points. Two weeks after implantation, Dkk3-loaded TEVGs display an increase of α-SMA+ cells, compared with control. Over time, the smooth muscle becomes more mature in Dkk3-TEVGs than in control grafts, as the area of SM-MHC+ cells is also increased. The images are representative of 4 grafts for each group. The data are expressed as the mean±SEM of 4 independent experiments. NS indicates nonsignificant. *P<0.05, compared with control group (2-way ANOVA followed by Bonferroni post hoc test for **B**, **E**, **F**, and **G**).

We then investigated whether Dkk3 could promote vascular cellularization and regeneration of the vascular grafts. Hematoxylin and eosin staining showed that the luminal diameter was markedly preserved in patent grafts without narrowing caused by intimal hyperplasia 3 months post-implantation (Figure 6A; Online Figure XII). Moreover, no significant difference in the inner diameter could be seen between Dkk3 and control groups at each time point (Figure 6B). DAPI (4,6-diamidino-2-phenylindole) staining revealed sufficient cell infiltration into the graft wall after 2 weeks, which is crucial for the following vascular regeneration (Online Figure XIII).^3^ After 3-month implantation, electron microscopy images throughout the vessel graft revealed that grafts loaded with Dkk3 were covered by a nearly confluent layer of neotissue, uniformly aligned along the blood flow, with a more homogeneous organization, when compared with control group (Figure 6C).

The effect of Dkk3 on vascular smooth muscle regeneration was evaluated by immunofluorescence staining using α-SMA (α-smooth muscle actin) and SM-MHC (smooth muscle myosin heavy chain) antibodies and subsequent measurement of the total area of α-SMA+ and SM-MHC+ cells in the luminal region in control and treated grafts. Two weeks post-implantation, Dkk3-treated grafts showed a significantly greater SMA+ area within the lumen region compared with the untreated grafts, but no difference could be measured anymore between the 2 groups 1 month post-implantation, indicating that Dkk3 promoted the recruitment of α-SMA+ cells in the graft at an early stage (Figure 6D and 6E). Interestingly, at later stages, that is, 1 (*P*<0.01) and 3 (*P*<0.005) months post-implantation, the SM-MHC+ area within the lumen region was significantly higher in the Dkk3 group in comparison with the control group (Figure 6D and 6F). These results demonstrated that Dkk3 promoted vascular regeneration by increasing the proportion of mature and functional SMCs, while reducing the overproliferation of SMCs that may lead to adverse intimal hyperplasia. Dkk3-TEVGs exhibited increased expression of SM-MHC and calponin and a reduced expression of α-SMA, in comparison with control TEVGs (Online Figure XIVA). Double immunofluorescence staining of α-SMA and SM-MHC further demonstrated that Dkk3 induced the transition of SMCs toward a contractile phenotype, exhibiting the typical spindle-shaped morphology (Online Figure XIVB).

Graft endothelization was analyzed by immunofluorescence staining with vWF (von Willebrand factor) antibody. ECs were first observed 2 weeks post-implantation in both groups. After 3 months of implantation, the vWF+ cell layer in the Dkk3 group appeared more uniform and continuous, characteristics of a more mature endothelium composed of a thin single sheet of ECs (Online Figure XV). These results are consistent with a recent study published by our group, which shows that Dkk3 accelerated re-endothelization in a femoral artery wire injury mouse model, confirming the role of Dkk3 on vascular endothelium integrity and function.^19^ Furthermore, double immunofluorescence staining of vWF and α-SMA also revealed a better tissue organization in Dkk3 group compared with the control group, as shown by the several layers of SMCs strategically aligned beneath a uniform layer of neoendothelium (Online Figure XVI). Concomitantly, the detection of extracellular matrix proteins, including elastin and collagen by Masson and Verhoeff-Van Gieson staining, showed a significant increase in the deposition of extracellular matrix content in the Dkk3 group 1 and 3 months post-implantation (Online Figure XVII). Taken together, these results show that Dkk3 enhances TEVG cellularization and remodeling—important processes involved in blood vessel maturation.

### Dkk3 Increases Sca-1+ Progenitor Cell Migration Through CXCR7 Activation and Contributes to Vascular Smooth Muscle Regeneration

**Figure 7.**
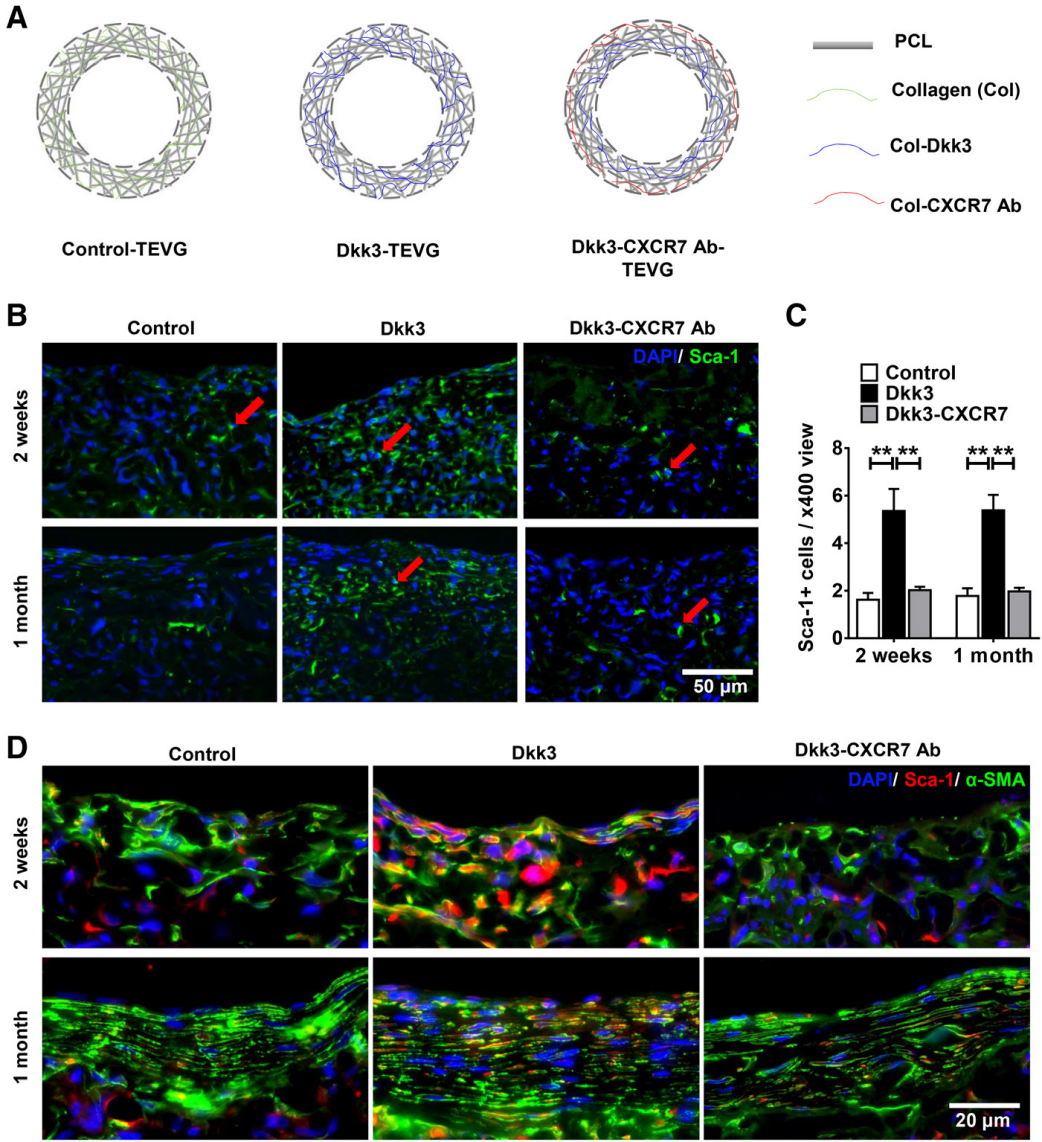
**Dkk3 (dickkopf-3) increases Sca-1+ progenitor migration and smooth muscle differentiation.** Schematic illustration of the 3 types of tissue-engineered vessel grafts (TEVGs). Control TEVGs composed of polycaprolactone (PCL) and collagen fibers; Dkk3-TEVG composed of PCL and Dkk3-collagen fibers; Dkk3-CXCR7 ab-TEVG composed of PCL, Dkk3-collagen, and CXCR7 antibody collagen fibers. **B**, Representative immunofluorescence images of the cross sections of TEVGs displaying Sca-1+ progenitor cells recruitment into the grafts detected with anti–Sca-1 (stem cell antigen-1) antibody (green), at the time points indicated. **C**, Quantification of Sca-1+ progenitor cell infiltration into the 3 types of TEVGs. DKK3 delivery significantly promotes the infiltration of Sca-1+ progenitor cells into the grafts. The incorporation of CXCR7 antibody in the grafts markedly inhibits infiltration of Sca-1+ progenitor cells into the grafts. **D**, Representative immunofluorescence images of the cross sections of TEVGs exhibiting double immunostaining with anti–α-SMA (α-smooth muscle actin; green) and anti–Sca-1 (red) antibodies, at the time points indicated. The increased contribution of double-positive cells (Sca-1+/α-SMA+) to the smooth muscle regeneration in Dkk3-TEVGs, compared with control group, seems to decrease in Dkk3-CXCR7 ab-TEVGs. DAPI was included to counterstain the nuclei. The data presented are representative images of 4 grafts for each group and are expressed as the mean±SEM of 4 independent experiments. **P<0.01, compared with control group (2-way ANOVA, followed by Bonferroni post hoc test).

Next, the specific effect of Dkk3 on Sca-1+ progenitor cells was further evaluated in our graft model, and the result indicated that incorporation of Dkk3 in the TEVGs remarkably enhanced the infiltration and maintenance of Sca-1+ stem/progenitor cells within the graft wall (Figure 7B and 7C). Furthermore, double immunofluorescence staining of α-SMA and Sca-1 (Figure 7D) showed a greater number of double-positive cells in Dkk3-loaded TEVGs compared with control group, indicating that some mobilized Sca-1+ cells could directly differentiate into SMCs and participate in the regeneration of vascular smooth muscle. To confirm the role of CXCR7 receptor in the Dkk3-driven migration of Sca-1+ progenitor cells, double-layered vascular grafts were designed and evaluated in in vivo models. In detail, in addition to the Dkk3-loaded inner layer, a CXCR7 antibody-loaded layer was introduced at the outside of the graft (Figure 7A). Because of the antagonistic effect provided by the CXCR7 antibody, the number of infiltrated Sca-1+ cells was dramatically reduced (Figure 7B and 7C), confirming that the chemotactic role of Dkk3 is dependent on CXCR7 activation. Furthermore, blocking of CXCR7 decreased the Dkk3-induced number of cells double-positive for Sca-1+ and α-SMA cells infiltrated into the grafts 2 weeks and 1 month post-implantation (Figure 7D). The effect of CXCR7 blocking in the grafts was also assessed for ECs, SMCs, and macrophages. The increased α-SMA+ area in Dkk3-TEVGs was reduced on CXCR7 blocking 2 weeks and 1 month post-implantation (Online Figure XVIIIA and XVIIIB). Likewise, SM-MHC+ area was also decreased in Dkk3-TEVGs when CXCR7 was blocked (Online Figure XVIIIA and XVIIIC). Regarding the ECs, 1 month post-implantation, although no difference was observed between control and Dkk3 groups in the endothelial coverage of the grafts, CXCR7 blocking decreased the graft endothelial coverage in the Dkk3-TEVGs (Online Figure XIX). Finally, no difference was detected in the recruitment of macrophages between control and Dkk3 groups or when CXCR7 blocked, 1 month post-implantation (Online Figure XX). Interestingly, at 1 month post-implantation, Dkk3 does not change in vivo the proliferation rate of ECs, SMCs, and macrophages (Online Figure XXI). Sdf-1α-TEVGs were also fabricated to further assess whether CXCR7 acts as a regulator of regeneration. 2 weeks post-implantation, similarly to Dkk3, Sdf-1α also induced the recruitment of Sca-1+ VPCs into the grafts, which was reduced on CXCR7 blocking (Online Figure XXII). Interestingly, macrophage recruitment was higher in Sdf-1α-TEVGs than in control TEVGs, and CXCR7 blocking decreased the Sdf-1α–induced migration of macrophages. On the contrary, no differences were observed between control and Sdf-1α groups in the recruitment of SMCs and in the endothelial coverage of the grafts. These results confirm that Dkk3-CXCR7 axis is crucial in mediating Sca-1+ cell migration and graft cellularization and regeneration in vivo.

## Discussion

Previous studies have reported that Sca-1+ progenitor cells are able to migrate and differentiate into a variety of cells and participate in vascular repairing and remodeling.^13,28,29,41^ In this study, we demonstrated that Dkk3 induces chemotaxis for Sca-1+ stem/progenitor cells via binding specifically to CXCR7 resulting in better regeneration of TEVGs. These conclusions are based on the following observations. First, Dkk3 can induce vascular progenitor migration in vitro in both transwell and wound healing assays and ex vivo in organ 3-dimensional culture assays using Sca-1-GFP aortic rings. Second, Dkk3 physically interacts with CXCR7 as indicated by coimmunoprecipitation and His-tag pull-down analysis, ligand-receptor affinity binding assays, artificial CXCR7-expressing system chemotaxis assay, and receptor blocking assays. Third, downstream signaling pathways mediated by Dkk3-CXCR7 binding were identified and confirmed using chemical inhibition and functional migration assays. Finally, Dkk3-loaded engineered vessel grafts displayed enhanced cellularization in vivo that could be blocked by application of anti-CXCR7 antibodies. Thus, the discovery of a new functional role for Dkk3 and the identification of its functional receptor could provide the basic information for the development of new drugs for vascular disease.

Chemokines are a class of cytokines that direct cell migration by binding to their corresponding chemokine receptor present on target cell types. The most important question is whether the chemotaxis of stem/progenitor cells is activated via binding of Dkk3 to a chemokine receptor. The chemokine receptors profile on Sca-1+ cells has been previously investigated through a systematic polymerase chain reaction array analysis, and the result showed that CXCR7 expression was remarkably higher than the expression of all the other receptors.^14^ Because CXCR7 is a receptor of Sdf-1α (a well-known chemokine with an established role in inducing chemotaxis of various cell types, including SMCs, ECs, and Sca-1+ VPCs^28,29,42,43^), we investigated whether CXCR7 could be the receptor of Dkk3 considering the similarity in the chemotactic ability between Dkk3 and Sdf-1α. In line with published data, our results confirmed that most Sca-1+ cells considerably expressed CXCR7, whereas the expression of the other receptors of Sdf-1α, such as CXCR4, was significantly low. Interestingly, as reported for Dkk3, CXCR7 expression is also upregulated in several cancers and tumor associated vasculature, which confers a proangiogenic role to CXCR7.^44,45^ Originally, CXCR7 was recognized as a mere scavenger receptor with the function to generate gradients of Sdf-1α.^46,47^ More recently, it is accepted as a functional receptor directly involved in cellular responses, including cell migration.^33,36,48–50^ In this study, knockdown of CXCR7 abrogated Dkk3-driven migration of progenitors, whereas CXCR7 overexpression increased Dkk3-mediated cell migration. Our data revealed that CXCR4 inhibition had no effect in Dkk3- or Sdf-1α–mediated migration of Sca-1+ cells, which indicated that CXCR4 was not implicated in this process. These results were consistent with previous studies that showed that CXCR4 antagonism with AMD3100 did not always inhibit Sdf-1α–induced migration because the chemokine acted via CXCR7.^50,51^ Collectively, our results demonstrate that CXCR7 is involved in Dkk3-driven migration of Sca-1+ cells.

Our data demonstrated that Dkk3 binds to CXCR7 with high affinity and in a specific, dose-dependent and saturable manner. The strength of their binding affinity was comparable with the interactions between Sdf-1α and CXCR7 or CXCR4, which were consistent with the reported data.^49,52^ Remarkably, our results are also in accordance with the findings described by Mao et al^22, 24^ that, contrarily to Dkk1 and Dkk2, Dkk3 does not bind to Kremen1 and Kremen2. Our findings provide the direct evidence of physical binding of Dkk3 to CXCR7, which may serve as a Dkk3 receptor.

**Figure 8.**
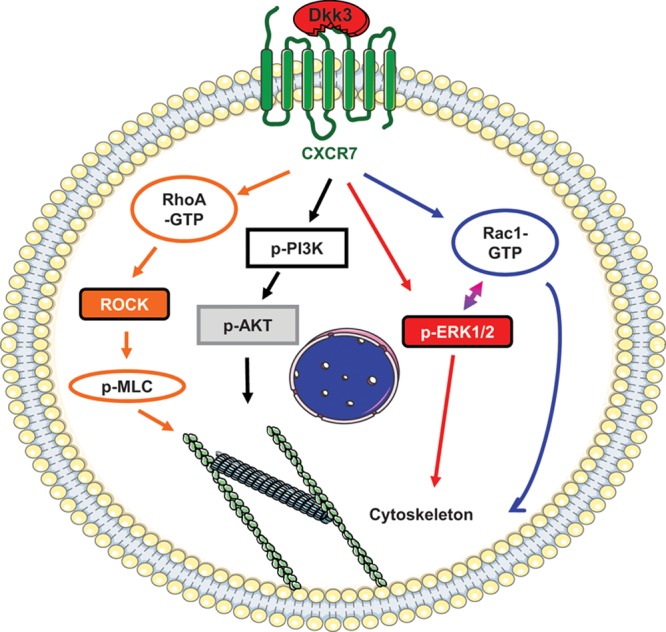
**Schematic illustration of Dkk3 (dickkopf-3)/CXCR7-mediated Sca-1+ progenitor migration.** The figure depicts the molecular mechanism involved in Sca-1 (stem cell antigen-1)+ progenitor migration induced by Dkk3 binding to CXCR7. Dkk3 present in the cell environment binds to CXCR7 expressed on the surface of Sca-1+ progenitors. On CXCR7 activation, ERK1/2 (extracellular signal-regulated kinases 1/2)-signaling pathway is activated. Phosphorylated ERK1/2 either activates Rac1 (Ras-related C3 botulinum toxin substrate 1) or directly modulates cytoskeleton rearrangement to promote cell migration. Rac1 activation also results from Dkk3 binding to CXCR7, which in turn directly regulates cell migration or stimulates ERK phosphorylation. Rac1 and ERK1/2 seem to work in a feedback manner. The Dkk3/CXCR7 axis promotes AKT phosphorylation via PI3K (phosphatidylinositol 3-kinase), which leads to cytoskeleton rearrangement. RhoA (Ras homolog gene family, member A) activation level is increased in response to Dkk3 binding to CXCR7, leading to MLC (myosin light chain) phosphorylation either directly or via ROCK (Rho-associated protein kinase) activation. MLC activation results in actomyosin contraction required for cell migration.

Among the multiple molecular mechanisms that regulate cell migration, we found that ERK1/2, PI3K/AKT, Rac1, and RhoA signaling cascades were involved in Sca-1+ VPCs migration induced by Dkk3. Dkk proteins are classically associated with the Wnt-signaling pathway, which comprises the canonical (β-catenin dependent) and the noncanonical (β-catenin independent) pathways.^39,40^ Unlike the other members of the dickkopf family that are Wnt/β-Catenin pathway antagonists,^39,40^ Dkk3, according to specific cell context, can act either as an agonist or inhibitor of the canonical Wnt signaling,^25,53^ or even exerts its effect independent of the Wnt pathway.^19,24^ Our findings show that none of the Wnt-signaling pathways is implicated in the migration mechanism of Sca-1+ cells stimulated by Dkk3. Noticeably, Sdf-1α induced the same migration molecular mechanisms. This result, allied with the fact that Dkk3 is the most divergent member of the Dicckopf family by several criteria,^40,54^ further supported our hypothesis of the chemotactic role of Dkk3 in stem/progenitor cells through CXCR7 activation. Finally, the molecular mechanisms involved in Dkk3-driven Sca-1+ cell migration are summarized in Figure 8.

Encouraged by the chemotactic properties of Dkk3 demonstrated from in vitro and ex vivo assays, we further assessed the role of Dkk3 in the migration of Sca-1+ progenitor cells in vivo and evaluated its potential to harness endogenous vascular regeneration by using a TEVG model. Dkk3-loaded tissue-engineered grafts effectively promoted the infiltration of Sca-1+ progenitor cells into the grafts, which was abrogated after the incorporation of CXCR7 antibody. Consistent with our results, studies have reported that Sdf-1α–loaded vascular grafts display an increased recruitment of endogenous progenitor cells and accelerate vascular regeneration. Additionally, these cells expressed CXCR7 and appeared to be progenitors of endothelial and SMCs.^2^ Our results also confirm the similarity between Dkk3 and Sdf-1α in terms of chemotactic function and establish the Dkk3-CXCR7 axis as regulator of regeneration.

An intact EC monolayer and the presence of mature SMCs in the media is vital in preventing thrombogenesis and maintaining long-term patency of vessel grafts.^55^ The ability of Dkk3 to promote re-endothelialization has been demonstrated in different models.^19,56,57^ Our study revealed that DKK3 promoted endothelialization in TEVGs, which exhibited a more uniform and organized alignment of ECs along the blood flow. Vascular cellularization and remodeling, which relies on the generation of a functional tunica media, is a critical aspect in TEVGs. Previous studies have demonstrated that Sca-1+ stem/progenitor cells can differentiate into SMCs under the stimuli of various cytokines,^13,28,29,41,58^ including Dkk3.^16,17,59^ In this study, Dkk3-loaded TEVGs exhibited a greater number of Sca-1+ cells, which coexpressed α-SMA. In vivo blocking of CXCR7 leads to a decreased number of SMCs present in the TEVGs, which may be because of the preceding reduction in the recruitment of VPCs into the TEVGs, which have the ability to migrate and differentiate into SMCs promoted by Dkk3 stimulation. More importantly, the SMC phenotype in Dkk3-TEVGs switched to a more mature and contractile phenotype in comparison with control TEVGs. Recently, we published a study proving the ability of Dkk3 to induce the differentiation of Sca-1+ VPCs into SMCs.^59^ The neovessel of Dkk3-loaded TEVGs exhibited a better tissue organization in structure with several SMC layers aligned beneath the endothelium, thus resembling that of native arteries. Therefore, our data show that Dkk3, through CXCR7, promoted better restructuration of the TEVGs and communication between the endothelial and SMC layers, which potentially contributes to a better endothelization of the vessel. Additionally, Dkk3 release over time increased the production of extracellular matrix, which is essential to provide the required mechanical strength to withstand the arterial pressure. The deposition of elastin and collagen was associated with the maturation of the smooth muscle layer, which supported the role of Dkk3 in promoting vascular graft arterialization. These findings are consistent with a recent study that reports that Dkk3 not only induces the differentiation of Sca-1+ VPCs into SMCs but also increases the content in SMCs and extracellular matrix deposition in atherosclerotic plaques, thus having a plaque stabilizing effect.^59^ The functionality of the TEVGs has been assessed previously.^60^ After 12 months of implantation, our TEVG grafts contracted and relaxed appropriately in response to the respective stimulants, thus proving that regenerated contractile SMCs were present in the grafts, which displayed a good vasoactivity, although still not at the level of the native vessel because of the slow degradation of the graft material. Furthermore, at 12-month implantation, all the grafts were patent, with absence of calcification, aneurism, stenosis, or harmful remodeling and exhibited rapid endothelization. Considering the ability of Dkk3 to induce an increase in the mature SMC pool and extracellular matrix deposition in the grafts when compared with control, we would speculate that the functionality and regeneration of the Dkk3-TEVGs would be at least similar to or better than the control TEVGs. Nevertheless, further investigation is required to clarify this issue.

The precise origin of the Sca-1+ progenitor cells present in the Dkk3-TEVGs is still under investigation. Published studies indicate that the Sca-1+ cells could originate from 2 sources, which are local resident and circulating progenitor cells.^8,13,41,61,62^ For the current study, we speculate that both sources may contribute to the pool of Sca-1+ cells in the Dkk3-TEVGs. We have shown in another study that a large number of cells infiltrating our TEVGs derives from the surrounding native tissue because the infiltrating cells migrate from the outer layers of the TEVGs to the inner layers.^3^ The use of Sca-1 knockin inducible Cre animal models would help us clarify the fate and progeny of Sca-1+ VPCs. Rigorous lineage-tracing methodology may further elucidate the role of Dkk3 in the migration, proliferation, and differentiation of Sca-1+ progenitor cells.

In summary, our work shows for the first time that Dkk3 plays a chemotactic role in VPCs through binding to the chemokine receptor CXCR7 and activating relevant downstream molecular mechanisms. Furthermore, the in vivo chemotactic property of Dkk3 and the potential to promote in vivo vascular graft arterialization have been verified in an animal model. Our findings herein presented provide evidence of the potential of Dkk3 and its receptor CXCR7 as targets for vascular drug-targeted therapy strategies and regenerative medicine.

## Sources of Funding

This work was supported by the British Heart Foundation (RG/14/6/31144), National Natural Science Foundation of China (No. 81522023, 81530059, and 91639113) and European Union’s Seventh Framework Programme for research, technological development, and demonstration (606998).

## Disclosures

None.

## Supplementary Material

## Nonstandard Abbreviations and Acronyms

α-SMA: α-smooth muscle actin
AP: alkaline phosphatase
ApoE: apolipoprotein E
CD: cluster of differentiation
CXCR: C-X-C chemokine receptor type
Dkk3: dickkopf-3
Dvl-PDZ: dishevelled protein with peptide Pen-N3
EC: endothelial cell
ERK1/2: extracellular signal-regulated kinases 1/2
GFP: green fluorescent protein
LRP: low-density lipoprotein receptor–related protein
MLC: myosin light chain
PI3K: phosphatidylinositol 3-kinase
Rac1: Ras-related C3 botulinum toxin substrate 1
RhoA: Ras homolog gene family, member A
ROCK: Rho-associated protein kinase
Sca-1: stem cell antigen-1
Sdf-1α: stromal cell-derived factor 1α
SM-MHC: smooth muscle myosin heavy chain
SMC: smooth muscle cell
TEVG: tissue-engineered vessel graft
VPC: vascular progenitor cell
vWF: von Willebrand factor

## Novelty and Significance

### What Is Known?

- Dkk3 (dickkopf-3) is involved in vascular remodeling, for example, atherosclerosis, vascular injury-induced stenosis, and plaque stability.
- Tissue-engineered vessel grafts constitute an effective alternative to the limited availability of autografts used for blood vessel replacement.
- Vascular progenitor cells play an active role in vascular remodeling and regeneration.
