# Supplementary material for: Binding of Dickkopf-3 to CXCR7 Enhances Vascular Progenitor Cell Migration and Degradable Graft Regeneration
Source: Circ Res. 2018 Jun 6;123(4):451–66. doi: 10.1161/CIRCRESAHA.118.312945 (PMC6092110; doi:10.1161/CIRCRESAHA.118.312945)
Supplement: Supplementary file 1 [file res-123-451-s001.pdf]

Supplemental Material

Methods:

Animals

All animal procedures were approved by the Institutional Committee for Use and Care of Laboratory Animals from UK Home Office and by the Center of Tianjin Animal Experiment Ethics Committee and Authority for Animal Protection. C57BL/6J mice were purchased from Harlan, UK. ApoE-deficient ($ApoE^{-/-}$) and Ly6a-GFP (Sca-1-GFP) transgenic mice were purchased from The Jackson Laboratory, USA. To generate transgenic Sca-1-GFP-$ApoE^{-/-}$ mice, Ly6a-GFP mice were crossed with ApoE-deficient mice. Sprague Dawley rats (10-12 week-old, 280-320g; both male and female about 50/50%) were purchased from Laboratory Animal Center of the Academy of Military Medical Sciences, Beijing, China. Animals were randomly grouped for treated and untreated controls.

Reagents and Antibodies

Pharmacological inhibitors PD98059, LY294002, AKT inhibitor X, Y27632, Rhosin, β-Catenin/Tcf inhibitor (FH535) and Dvl-PDZ Domain inhibitor were all purchased from Calbiochem/Millipore. NSC23766 inhibitor was acquired from Sigma-Aldrich. Poly(ε-caprolactone) (PCL) pellets (average M_n=80,000) were purchased from Sigma-Aldrich (St.Louis, USA). Type-I collagen was kindly donated by Sannie Bioengineering Technology Company (Tianjin, China). DKK3 (1118-DK) and primary antibody CXCR7 (MAB8399) for vascular graft loading were obtained from R&D (USA). Sdf-1α (400-32A-50) was purchased from Peprotech (USA). Hexafluoroisopropanol (HFIP) was purchased from Aladdin Chemistry Co. (China). All other chemicals were obtained from Tianjin Chemical Reagent Company (Tianjin, China). For immunofluorescence staining of the vascular grafts antibodies against α-SMA (ab7817), SM-MHC (ab681), vWF (ab6994), CD31 (ab64543), Ki67 (ab16667) and CXCR7 (ab72100) were purchased from Abcam; the antibody against Sca-1 (AB4336) was purchased from Millipore (Germany) andthe antibody against vWF (A0082) was purchased from Dako (USA). All secondary antibodies were obtained from Invitrogen (USA). For *in vitro* studies, Dkk3 recombinant mouse protein, His tag (50247-M08H-50) was obtained from Thermo Fisher and Sdf-1α recombinant murine protein (250-20A) was acquired from PeproTech. For Western blot analysis the antibodies p-ERK 1/2 (Thr202/Tyr204) (sc-16982), t-ERK 1/2 (sc-135900), p-AKT 1/2/3 (Ser473) (sc-7985-R), t-AKT 1/2/3/ (sc-8312), CXCR4 (sc-6190), β-Catenin (sc-7199) and GAPDH (sc-25778) were purchased from Santa Cruz Biotechnology; p-MLC 2 (Thr18/Ser19) (3674) and t-MLC 2 (3672) were acquired from Cell Signalling Technology; Axin-2 (ab32197) was purchased from Abcam; CXCR7 (NBP1-31309) was purchased from Novus Biologicals and Dkk3 was obtained from GenScript. Secondary antibodies were either acquired from Dako or LI-COR Biosciences. For Co-IP assay, CXCR7 antibody (NBP1-31309) and respective Rabbit IgG Isotype Control (NB810-5610) were obtained from Novus Biologicals. The antibodies used in flow cytometry included the conjugated antibodies Sca-1-PE-Cy7 (BD Biosciences, 558162) and CXCR4-PE (R&D, FAB21651P), the primary antibody CXCR7 (NBP1-31309) and the secondary antibody Alexa Fluor® 647 (ThermoFisher). The Anti-HA tag antibody (ab9110) used in affinity binding assay to coat the ELISA plate was purchased from Abcam and

recombinant human proteins Dkk3 (1118-DK-050) and Sdf-1α (350-NS-010/CF) were purchased from R&D.

Sca-1+ Adventitial Progenitor Cell Isolation, Sorting and Culture

Mouse vascular progenitor cells were isolated from the outgrowth of aortic adventitial tissues as previously described[1]. Briefly, aortic arch and root from $ApoE^{-/-}$ mice were harvested under sterile conditions. The adventitia was carefully detached from the media and intima layers, cut into pieces and seeded onto gelatin-coated flask. Following attachment onto the flask, complete stem cell medium (DMEM ATCC 30-2002, 10% ES Cell Qualified Fetal Bovine Serum (Embriomax), 10ng/mL of Leukemia inhibitor factor (LIF), 0.1mM β-Mercaptoethanol, 100U/mL Penicillin/Streptomycin and 2mM L-glutamine) was added for 5-7 days. After reaching 90% confluency, the cells were selected for the Sca-1+ marker using the Anti-Sca-1 MicroBead kit (Miltenyi Biotec) and cultured in complete stem cell medium. The passage numbers of the cells used in the experiments were between passages 5 and 15.

Fabrication and Characterization of Tissue-Engineered Vascular Grafts (Tevgs)

The hybrid fibrous scaffolds were fabricated by co-electrospinning. A homogeneous PCL solution (25%, w/v) was obtained by dissolving PCL pellets in methylene chloride and methanol (5:1, v/v) mixture. Type-I collagen was dissolved in Hexafluoroisopropanol (HFIP) at a concentration of 8% (w/v) solution, and then it was mixed with BSA (5mg/mL) and DKK3 (200μg/mL) or Sdf-1α (200μg/mL) solutions at ratio of 4:1:1 (v/v/v) under constant stirring overnight at room temperature[2]. PBS was used instead of Dkk3 or Sdf-1α solution in the control group. The vascular grafts were fabricated by co-electrospining PCL and collagen solutions onto a stainless-steel rod (D=2mm) as the collector according to the following processing parameters: the needle tip-collector distances were 25 and 16cm; the flow rates were 8 and 0.6 mL/h; the voltages were set as 11kV and 17kV with a 21-G needle for PCL and collagen, respectively. The bi-layered grafts were prepared with the same processing parameters. Dkk3 (200μg/mL) or Sdf-1α (200μg/mL) and CXCR7 (400μg/mL) loaded collagens were used to prepare the inner and outer layers respectively, with a volume ratio of 5:1. The as-prepared grafts were dried under vacuum at room temperature for more than 3 days to sufficiently remove the residual solvents, followed by exposition to UV light overnight before implantation[3]. The structure of the electrospun vascular grafts was analysed using a scanning electron microscope (SEM) (Quanta200, Czech). To determine the fiber diameter, six SEM images were analysed, and at least 10 fibers were manually measured on each image using Image J software (NIH USA, 2008). The measurement of Dkk3 release from the grafts was performed by immersing the weighted grafts (10mg) into 1mL of phosphate-buffered saline (PBS) buffer at 37°C. At each time point, supernatants were collected and replaced by 1mL of fresh PBS. The concentration of Dkk3 in the supernatant was determined using Dkk3 ELISA kit (DY1118, R&D).

Implantation and Explantation of Vascular Grafts

Rats were anesthetised by intraperitoneal injection of chloral hydrate (300mg/kg). Heparin (100 Units/kg) was injected through the tail vein before surgery for anticoagulation. A midline laparotomy incision was performed before the abdominal aorta was isolated, clamped, and transected. The vascular grafts (2.0 mm in inner diameter and 1.0 cm in length) were sewed in an end-to-end fashion with 8 interrupted stitches using 9-0 monofilament nylon sutures

(Chenghe, Jiangsu, China). The skin was sewed with 3-0 monofilament nylon sutures. No anticoagulation drug was administered to the rats post-surgery. The patency of the implanted grafts was determined by examining the blood flow in the blood vessel using the Color Ultrasound Doppler (Vevo 2100 System, Canada), after the rats were anesthetised with isoflurane. The graft was defined as being patent only if normal blood flow, lumen free of occlusion or thrombus and absence of aneurism were observed. At predetermined time-points, animals were sacrificed by injection of overdose chloral hydrate and the vascular grafts were explanted, and rinsed with saline. Micro-CT (Quantum FX) analysis was performed to detect the calcification in the explanted grafts. Then the samples were cut into two parts from the middle. One part was fixed with 4% paraformaldehyde and embedded in optimal cutting temperature (OCT) for frozen cross-section. The other part was longitudinally cut into two pieces. One piece was first observed by stereomicroscope (LEICA S8AP0, Germany), and then fixed with paraformaldehyde for longitudinal section. The other piece was fixed with 2.5% glutaraldehyde overnight and then dehydrated in ascending series of ethanol for SEM examination. For animal studies, the surgical team members were blinded to all the groups. Analyses were performed by another investigators in a blinded fashion. No animals or samples were excluded from the analyses.

Aortic Ring Assay and Immunofluorescence Staining

Aorta vessels were isolated from wild type and $ApoE^{-/-}$ mice and washed with DMEM. The connective and fat tissue were separated and transverse cuts were made in order to obtain aortic rings of 1 mm in size. After washing with PBS, the explants were placed in Matrigel (Corning) and cultured in either control medium (1% FBS in stem cell ATCC medium) or treatment medium (control medium supplemented with either 25 ng/mL of Dkk3 or 25 ng/mL of Sdf-1α) for 8 days. Control and treatment media were replaced every other day. On day 8 cell outgrowth was quantified under phase contrast microscope (Nikon Eclipse TS100). 6 aortic rings were considered for each condition. For characterisation of the cell outgrowth, transgenic Sca-1-GFP-$ApoE^{-/-}$ mice were used. After 8-day cell outgrowth, the rings were fixed (2% PFA and 0.1% glutaraldehyde in PBS) and permeabilised (0.1% Triton X-100). Following staining with DAPI, the images were acquired using Nikon A1R Confocal microscope and processed using ImageJ software. Sca-1+-GFP cell quantification was performed by counting all the GFP+ cells in the outgrowth and by determining the percentage of Sca-1+-GFP cells against the total number of cells (DAPI) present in the outgrowth.

Transwell Migration Assay

Sca-1+ VPCs were starved for at least 12 hours. The next day, in the lower chamber of transwell inserts with 8.0 µm pore size membrane filters (#3422, Corning Life Science, USA), 800 µL of control (0.2% FBS in ATCC medium) or treatment (0-100 ng/mL of Dkk3 or Sdf-1α in 0.2% FBS in ATCC medium) medium was added. In the upper chamber of the transwell insert, $5x10^4$ cells resuspended in 200 µL of control medium were loaded. After 12 hours of incubation, non-migrating cells on the upper chamber side of the filters were carefully removed using a cotton bud. The migrated cells (through the pores) on the lower side of the membrane were fixed with 4% PFA in PBS and stained with 0.1% crystal violet dye. Stained cells were counted in 5 different fields of each insert at 10X magnification (Nikon Eclipse TS100). For each condition duplicates were considered. Data was expressed as the fold of migrated Sca-1+ VPCs in response to treatment compared to control. For

experiments involving inhibitors, Sca-1+ cells were pre-incubated for 1 hour with the inhibitors prior to performing the transwell assay. Inhibitors were also added to the lower chamber of the inserts.

Wound Healing Assay

$1x10^4$ Sca-1+ VPCs were seeded in 12-well plates with complete medium until reaching confluency of 80%. Next, cells were starved for at least 6 hours and then washed with PBS. Using a clean 1000 μL pipette tip a straight scratch wound was made across the well. Cell debris was removed with PBS and then control (0.2% FBS in ATCC medium) or treatment (0-100 ng/mL of Dkk3 or murine Sdf-1α, in 0.2% FBS in ATCC medium) media were added to the wells. The cells were allowed to migrate (close the scratch/wound) for 12 hours. Subsequently, the medium was removed and the cells were washed with PBS, followed by fixation with 4% PFA in PBS. Finally, cells were stained with 0.1% crystal violet dye. After washing the wells with PBS, the number of migrated cell into the wound was quantified under the microscope at 10X magnification (Nikon Eclipse TS100), considering 5 random fields per scratch. Duplicates were used for each condition. Data was expressed as the fold of migrated Sca-1+ VPCs in response to treatment compared to control. For experiments involving inhibitors, these were added to the corresponding wells with the control or treatment medium.

Co-immunoprecipitation

Co-immunoprecipitation assay was performed according to the instructions provided in Pierce Co-immunoprecipitation Kit (ThermoFisher), with minor modifications: Sca-1+ VPCs were starved for at least 12 hours, after which they were treated with 25 ng/mL of Dkk3 for 3 hours. The lysate was pre-cleared by using the Control Agarose Resin column. Antibody immobilisation columns were prepared with rabbit CXCR7 antibody and rabbit IgG control. Sample lysate was incubated in the column overnight at 4°C. The eluted immune complexes and input samples were separated on a 4-12% Bis-Tris gel (NuPage®, Novex) and the immunoblot was probed with Dkk3 antibody.

His-tag Pull-down Assay

His-tag pull-down assay was performed according to the instructions provided in MagneHis Protein Purification System (Promega), with minor modifications. Dkk3-His tagged recombinant protein (25 ng/mL) was used to treat Sca-1+ VPCs for at least 3 hours. The cell lysate was incubated with Ni-particles as instructed. After washing, the eluate was used for immunoblotting. CXCR7 and CXCR4 antibodies were used to assess their binding to Dkk3.

Affinity Binding Assay

Receptor affinity binding assay was done as described previously[4]. In brief, white high-binding ELISA 96-well plate (Greiner) was coated overnight at 4°C with 100 μL/well of 2 μg/mL of anti-HA tag antibody (Abcam) in $NaHCO_3$ buffer (50 mM, pH 9.6). After washing the wells six times with TBS-T, blocking (5% BSA in TBS-T) was followed on a shaker for 1 hour at room temperature. Meanwhile, HEK 293T cells overexpressing hCXCR4, hCXCR7, hKremen1 and hKremen2, after washing with ice-cold PBS, were lysed with RIPA lysis and extraction buffer (Thermo Fisher) containing protease inhibitors. The cell lysate was diluted in blocking buffer. Next, 100 μL of membrane extract lysate was added to the corresponding HA-coated wells and allowed to bind overnight at 4°C on a shaker. Following extensive

washing, the wells were blocked for 1 hour at room temperature. The blocking buffer was removed and the diluted solutions of hDkk3-AP and hSdf-1α-AP were added to the wells for 2 hours of incubation at room temperature with gentle shaking. Next, the wells were washed and bound AP activity was measured using chemiluminescent SEAP Reporter Gene Assay (Roche). For each concentration of either hDkk3-AP or hSdf-1-AP, background (wells without cell lysate) binding value was subtracted. As a control, serially diluted AP alone was also added to the wells containing CXCR7 overexpressing cell lysate. Binding curve, Scatchard plot and dissociation constant (Kd) were obtained using GraphPad Prism 7 software.

Histological Analysis of The TEVG Explants

Samples prepared for SEM were first mounted onto aluminium stubs and then sputter-coated with gold. For sectioning and staining, the explants fixed with 4% paraformaldehyde were dehydrated by 30% sucrose solution until the grafts sank to the bottom. The explants were sectioned to 6 μm in thickness after being embedded in OCT. Subsequently, the sections were stained with haematoxylin and eosin (H&E), Masson's trichrome, and Verhoeff–van Gieson (VVG). Images were observed under upright microscope (Leica DM3000). For immunofluorescence staining, standard procedures were followed as previously described[5]. The smooth muscle cells were stained using α-SMA (1:100) and SM-MHC (1:100) primary antibodies. Endothelial cell staining was performed using vWF (1:200) primary antibody. The sections incubated with rat IgG were used as negative controls. Images were observed under a fluorescence microscope (Zeiss AxioImager Z1, Germany) and processed by Adobe Photoshop software.

Cell Proliferation Assay

The Sca-1+ cell proliferation assay was performed using the Cell Proliferation ELISA BrdU colorimetric Kit (Roche), according to manufacturer’s instructions. Cells were starved for at least 12 hours before treatment for 12, 24 or 48 hours. Absorbance was measured using a microplate ELISA reader (Infinite M200 PRO, Tecan).

Cell Death Assay

The analysis of the effect of Dkk3 in Sca-1+ cell death by apoptosis or necrosis was performed according to the instruction provided in the FITC Annexin V Apoptosis Detection Kit II (BD Biosciences). Sca-1+ cells were starved for at least 12 hours and then treated with Dkk3 (25 ng/mL) for 24 hours. Cell death rate was assessed at 1, 4, 8, 16 and 24 hours of stimulation by flow cytometry (BD Accuri™ C6).

Flow Cytometry Analysis

Sca-1+ cells were harvested and washed in PBS. After centrifugation, cell pellet was resuspended in blocking buffer (5% swine serum in PBS) and incubated for 30 minutes at 4°C. Following centrifugation, cells were incubated with CXCR7 primary antibody (5 μg/mL) or mouse IgG as a control for 2 hours at 4°C. Subsequently, cells were washed, centrifuged and secondary antibody Alexa Fluor 647 (APC) was added together with conjugated antibodies CXCR4-PE and Sca-1-PE-Cy7, with 1 hour of incubation at 4°C. Finally, cells were washed, centrifuged and resuspended in PBS. Fluorescence Minus One (FMO) samples were also prepared and compensation was performed. Samples were analysed

using the instrument BD Accuri™ C6. Data analysis was performed using the software FlowJo_V10.

RT-PCR

Total RNA was isolated and purified using the QIAGEN RNeasy Mini Kit according to manufacturer's instructions. 1 μg of RNA was reversely transcribed into cDNA using the QuantiTect® Reverse Transcription Kit (Qiagen).

Quantitative Real Time Polymerase Chain Reaction (QPCR)

Real Time PCR was performed using 20 ng of cDNA per sample with a SYBR Green Master MIX (PCR Biosystems) in a 20μL reaction. Ct values were measured using the Eppendorf Mastercycler® ep Realplex and GAPDH housekeeping gene was used as the endogenous control to normalize the amounts of RNA in each sample. The sequences of the primers are listed in Online Table I.

Western Blot Analysis

Cells with or without treatment were lysed using RIPA buffer with phosphatase and protease inhibitors. The lysate was sonicated (Branson Sonifier) prior to 30 minutes incubation on ice. Next, the samples were centrifuged and the supernatant was transferred to a new 1.5 mL tube. Protein concentration was measured using the Bradford method (Bio-Rad Smartspec™ Plus). 20-45 μg of protein lysate mixed with SDS loading buffer were applied to 4-12% Bis-Tris gels (NuPage, Novex) immersed in MOPS SDS running buffer (NuPage), before being transferred to a nitrocellulose membrane (Amersham Biosciences), followed by standard western blotting procedure.

G-LISA Small GTPase Activation Assay

The small GTPases activation assays were performed according to the instructions provided in RhoA and Rac1 G-LISA Activation Assay Kits, in the colorimetric format (Cytoskeleton Inc.). Sca-1+ VPCs were starved for at least 12 hours and in the next day, control (0.2% FBS in ATCC medium) and treatment (25 ng/mL of Dkk3 or Sdf-1α, in 0.2% FBS in ATCC medium) media were added to the cells. Upon reaching the desired time points, cells were washed and then lysed with ice-cold Lysis buffer. All samples were equalised as recommended. Absorbance was measured in the microplate reader Infinite M200 PRO (Tecan).

Knockdown of Target Genes by Sirna Transfection

Mouse CXCR7 SiRNA (MISSION esiRNA mouse CXCR7), Mouse Kremen1 SiRNA (Mission esiRNA mouse Kremnen1), Mouse AKT1 SiRNA (MISSION esiRNA mouse AKT1), Mouse AKT2 SiRNA (MISSION esiRNA mouse AKT2), and respective control (MISSION esiRNA EGFP) were purchased from Sigma-Aldrich. Mouse Makp1 SiRNA (siGENOME Mapk1) and respective control (siGENOME Non-Targeting) were obtained from Dharmacon (USA). Downregulation of the target genes was performed by siRNA transfection using Lipofectamine RNAiMAX (ThermoFisher). After 48 hours of transfection the cells were washed with PBS and then harvested for transwell migration assay and Western blot. The level of downregulation was assessed using real time qPCR and Western blot analysis.

Cloning of the Receptors

Human receptors CXCR4, CXCR7, Kremen1 and Kremen2 were cloned in a pShuttle-FLAG-HA vector (kindly supplied by Dr. Lingfang Zeng) using Nhe I and Sal I restriction enzymes (NEB Inc. Biolabs) in order to obtain HA tag in C-terminal. The cDNA and primers used to amplify the receptors are listed in Online Table II. After amplification of the receptors by conventional PCR, the inserts were separated by gel electrophoresis and extracted using the QIAquick Gel Extraction Kit (Qiagen). Following confirmation of the respective sequences, the receptor inserts and the vector were digested with the mentioned restriction enzymes and then separated from their reaction mixture components by gel electrophoresis. After gel extraction, the nucleic acid concentration and purity were assessed using the NanoDrop N-1000 spectrophotometer. Ligation was performed overnight at 14°C with T4 DNA Ligase (Promega), according to manufacturer's instructions. Finally, 100 ng of the resulting product were transformed in JM109 E. coli competent cells (Promega, L1001) and the transformed cells were spread in LB-agar plates containing Kanamycin. Selected colonies were amplified overnight and the receptor-HA plasmids were extracted using QIAprep Spin Miniprep Kit (Qiagen).

Luciferase Reporter Assay

M50 Super 8x TOPFlash and M51 Super 8x FOPFlash (TOPFlash mutant) were acquired from Addgene (Addgene plasmid # 12456 # 12457). Cells were seeded in 96-well plates. 24 hours later, transfection was performed with Lipofectamine 3000 (Invitrogen) according to the manufacturer's protocol. pRL-TK *Renilla* vector (Promega, E2231) was also included in all transfections as an internal control. 48 hours after transfection, the medium was changed to treatment medium containing either Wnt3a (100ng/mL), or Dkk3 (25 ng/mL), or PBS (Control). Luciferase activity was measured with the Dual-Glo Luciferase Reporter Assay System (Promega) 24 hours after treatment. The results were normalized against *Renilla* activity.

Receptor Overexpression in HEK 293T Cells

HEK 293T cells were seeded and once cell confluency reached 75%, the medium was changed to 1% FBS DMEM without any antibiotics. Plasmids were transiently transfected into HEK 293T cells in 6 well plates using Fugene® HD transfection reagent (Promega). After 48 hours of transfection, cells were harvested and receptor overexpression was analysed by real time qPCR.

Alkaline Phosphatase Conjugation

Human Dkk3 and Sdf-1α were reconstituted at concentrations of 200 μg/mL and 100 μg/mL, respectively. Alkaline Phosphatase (AP) was fused to each recombinant protein using the Lightening-Link® Alkaline Phosphatase Conjugation Kit (Innova Biosciences). The efficiency of AP conjugation was assessed using the NFkb Secreted Alkaline Phosphatase (SEAP) Reporter Assay Kit (Novus Biologicals). hDkk3-AP, hSdf-1α-AP and AP alone were serially diluted. AP activity was measured by reading the absorbance at 405 nm in the Infinite M200 PRO (Tecan) ELISA plate reader. The concentration of the conjugated-recombinant protein samples was calculated, and the efficiency of AP conjugation was determined by calculating the correlation between AP activity and hDkk3-AP or hSdf-1α-AP concentrations.

Statistical Analysis

The mean values ± s.e.m. were calculated and plotted using GraphPad Prism 7 software. Data distribution was evaluated by Shapiro-Wilk normality test. For normally distribution, Comparisons between two groups were performed using two-tailed Student's unpaired t-test. Differences between multiple groups with one variable were determined using one-way analysis of variance (One-way ANOVA), followed by Bonferroni's post-hoc test. To compare multiple groups with more than one variable, Two-way ANOVA, followed by Bonferroni's post-hoc test was used. Non-parametric Mann-Whitney test or Kruskal-Wallis ANOVA test were performed for data sets without evidence for normal distribution. $P<0.05$ was considered statistically significant.

References

1. Hu Y, Zhang Z, Torsney E, Afzal AR, Davison F, Metzler B, Xu Q. Abundant progenitor cells in the adventitia contribute to atherosclerosis of vein grafts in ApoE-deficient mice. *Journal of Clinical Investigation.* 2004;113:1258-1265.
2. Valmikinathan CM, Defroda S, Yu X. Polycaprolactone and bovine serum albumin based nanofibers for controlled release of nerve growth factor. *Biomacromolecules.* 2009;10:1084-1089.
3. Yao Y, Wang J, Cui Y, Xu R, Wang Z, Zhang J, Wang K, Li Y, Zhao Q, Kong D. Effect of sustained heparin release from PCL/chitosan hybrid small-diameter vascular grafts on anti-thrombogenic property and endothelialization. *Acta Biomater.* 2014;10:2739-2749.
4. Mao B, Wu W, Davidson G, Marhold J, Li M, Mechler BM, Delius H, Hoppe D, Stannek P, Walter C, Glinka A, Niehrs C. Kremen proteins are Dickkopf receptors that regulate Wnt/[beta]-catenin signalling. *Nature.* 2002;417:664-667.
5. Zhu M, Wang Z, Zhang J, Wang L, Yang X, Chen J, Fan G, Ji S, Xing C, Wang K, Zhao Q, Zhu Y, Kong D, Wang L. Circumferentially aligned fibers guided functional neoartery regeneration in vivo. *Biomaterials.* 2015;61:85-94.

Online Table I. Sequences of the primers used in quantitative real time PCR

Gene of interest	Forward primer sequence	Reverse primer sequence
Human CXCR4	5' - CAAGCAAGGGTGTGAGTTTG - 3'	5' - GGCTCCAAGGAAAGCATAGA - 3'
Human CXCR7	5' - TTCTCCTACGTGGTGGTCTT - 3'	5' - GCAGGTGAAAGGGATGTAGTG - 3'
Human GAPDH	5' - CATGTTCGTCATGGGTGTGAACCA - 3'	5' - AGTGATGGCATGGACTGTGGTCAT - 3'
Mouse Tcf1	5’ - GCCTGGTGTTGTATCAGAGT - 3’	5’ – GCCATCTGGGTGGAGATAAA - 3’
Mouse β-catenin	5’ - GACACCTCCCAAGTCCTTTATG - 3’	5’ - CTGAGCCCTAGTCATTGCATAC - 3’
Mouse Axin-2	5’ - CCTATGCCCGTTTCCTCTAATG - 3’	5’ - TCACCCAACAAGGAGTGTAAAG - 3’

Online Table II. Sequences of primers used in conventional PCR for cloning

Gene of interest	Forward primer sequence<br>Reverse primer sequence	Product size	cDNA source
Human CXCR4	5’-ATATGCTAGCATGTCCATTCCTTTGCCTC–3’<br>5’-ATATTCTAGAGCTGGAGTGAAAACTTGAAG–3’	1.011 kb	HUVEC
Human CXCR7	5’-ATATGCTAGCATGGATCTGCATCTCTTCGACTA–3’<br>5’–ATATTCTAGATTTGGTGCTCTGCTCCAAGG–3’	1.089 kb	HUASMC
Human Kremen1	5’ – ATATGCTAGCATGGCGCCGCCAGCCGCCCGC – 3’<br>5’ – ATATGTCGACGATACTTCTGGGCTGCCCCTG – 3’	1.479 kb	HG16100-G<br>(Sino Biological Inc.)
Human Kremen2	5’ – ATATGCTAGCATGGGGACACAAGCCCTGCAG - 3’<br>5’ – ATATGTCGAC GAGAGCGGAGATGAGCGAGCG – 3’	1.687 kb	MHS6278-202829187<br>(Dharmacon)

a

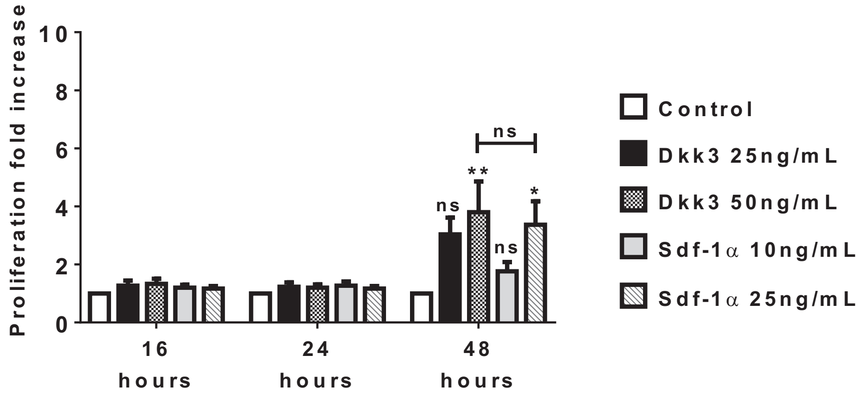

b

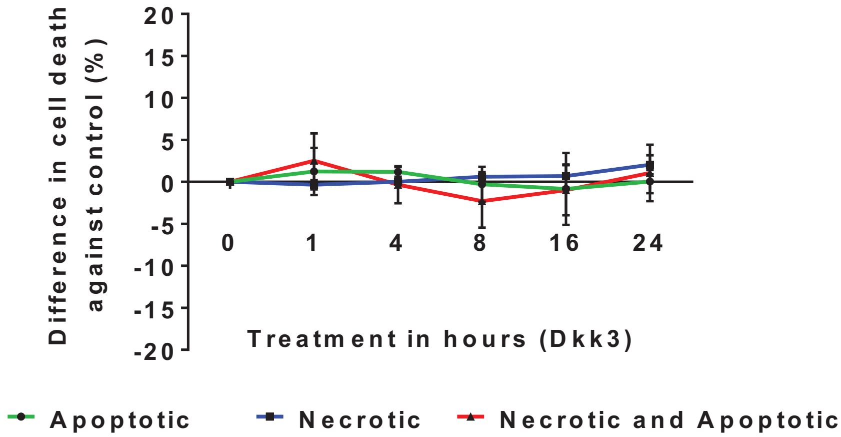

Online Figure I. The effect of Dkk3 on Sca-1+ cell proliferation and cell death *in vitro*. (**a**) The effect of Dkk3 and Sdf1-α on Sca-1+ cell proliferation rate was determined using the BrdU incorporation analysis. Sca-1+ cell proliferation rate is not induced by Dkk3 (25 ng/mL) or Sdf-1α (10 ng/mL) at 15, 24 and 48 hours of treatment. At higher concentration, both Dkk3 (50 ng/mL) and Sdf-1α (25 ng/mL) induce Sca-1+ cell proliferation only at 48 hours of stimulation. (**b**) Cell death by apoptosis and necrosis was evaluated using annexin V/Propidium Iodide staining and flow cytometry. The difference in the percentage of cell death between treatment (Dkk3 25 ng/mL) and control groups is shown over a period of 24 hours. Dkk3 treatment during 24 hours does not affect significantly the rate of Sca-1+ cell death by apoptosis or necrosis. The data are expressed as the mean±s.e.m. of 3 to 4 independent experiments. *P<0.05, ***P<0.001, compared with control group (0 ng/mL) (Two-way ANOVA followed by Bonferroni's post-hoc test).

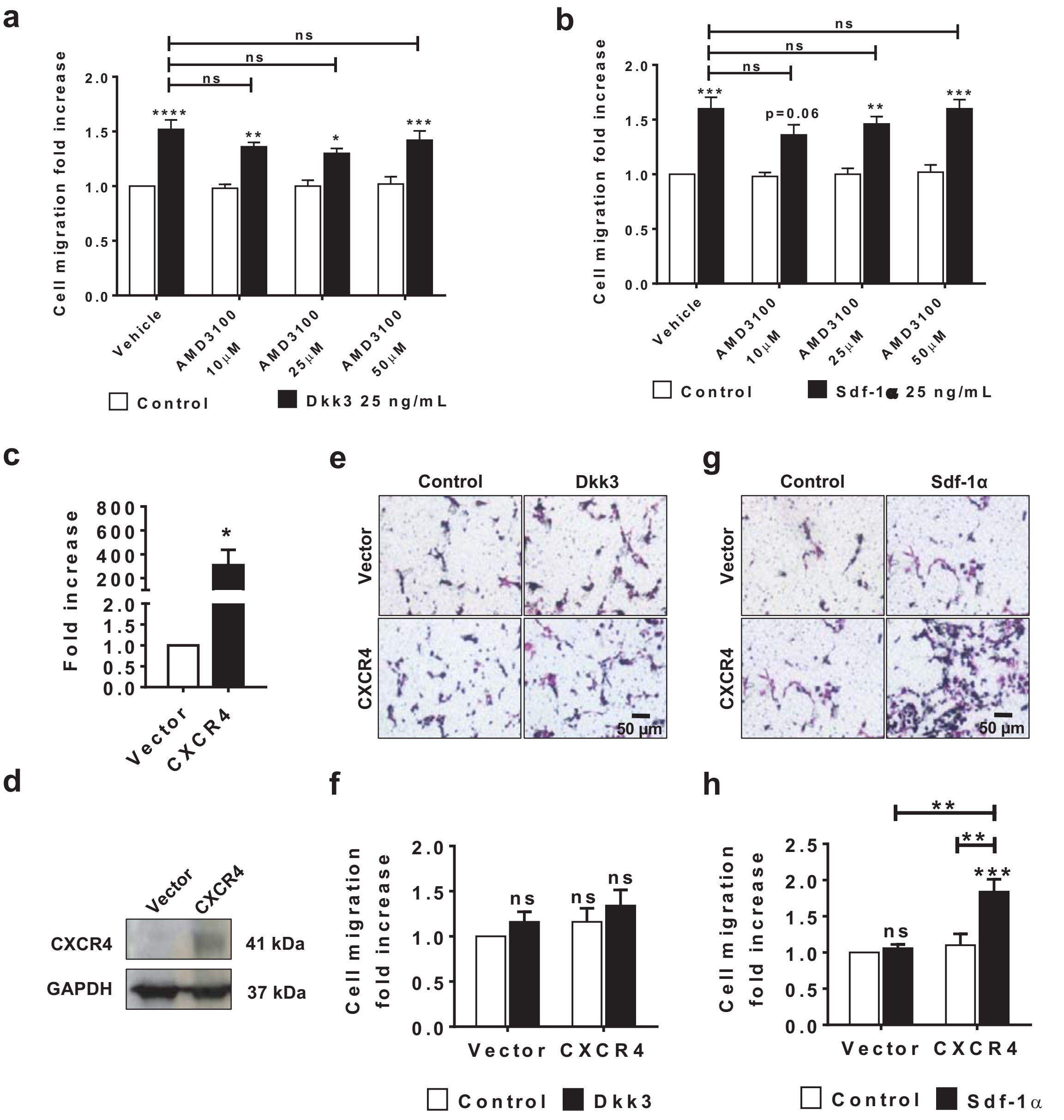

Online Figure II. CXCR4 is not involved in Sca-1+ cell migration. (**a**, **b**) Quantification of Sca-1+ cell migration driven by Dkk3 and Sdf-1α, respectively, in response to CXCR4 inhibitor AMD3100 (n=4). Neither Dkk3- or Sdf-1α-mediated migration of Sca-1+ cells is affected by CXCR4 inhibition. (**c**) Quantitative RT-PCR analysis of CXCR4 expression in HEK 293T cells transfected with CXCR4 expressing plasmid. Expression levels were normalized against GAPDH (n=5). (**d**) Western blot analysis of CXCR4 overexpression in HEK 293T cells transfected with CXCR4 expression plasmid. (**e, g**) Representative images of transwell migration assays of CXCR4 overexpressing HEK 293T cells stimulated with either Dkk3 or Sdf-1α (25 ng/mL) treatment. (**f, h**) Quantitative analysis of migrated cells in response to either Dkk3 or Sdf-1α treatment. While Sdf-1α induces the migration of CXCR4 overexpressing HEK cells, Dkk3 is not capable of promoting their migration (n=5). The data are expressed as the mean±s.e.m. of 4 to 5 independent experiments. *P<0.05, **P<0.01, ***P<0.001, compared with control group (0 ng/mL) (two-tailed unpaired Student's t-test for **c**; Two-way ANOVA followed by Bonferroni's post-hoc test for **a**, **b**, **g** and **h**).

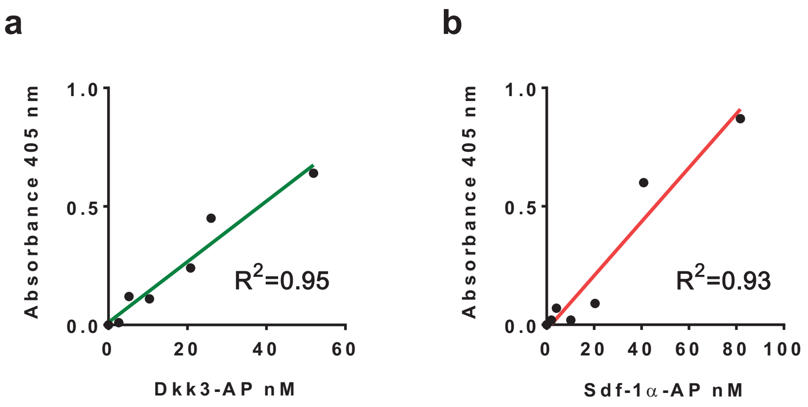

Online Figure III. Dkk3 and Sdf-1α human proteins are efficiently conjugated to Alkaline Phosphatase (AP). (**a**, **b**) Efficiency of conjugation of Alkaline Phosphatase (AP) with Dkk3 and Sdf-1α, respectively. The activity of AP was measured colorimetrically. AP activity increased linearly with Dkk3 and Sdf-1α concentration. The graphs shown for each ligand protein correspond to the one most representative, amongst 3 independent experiments.

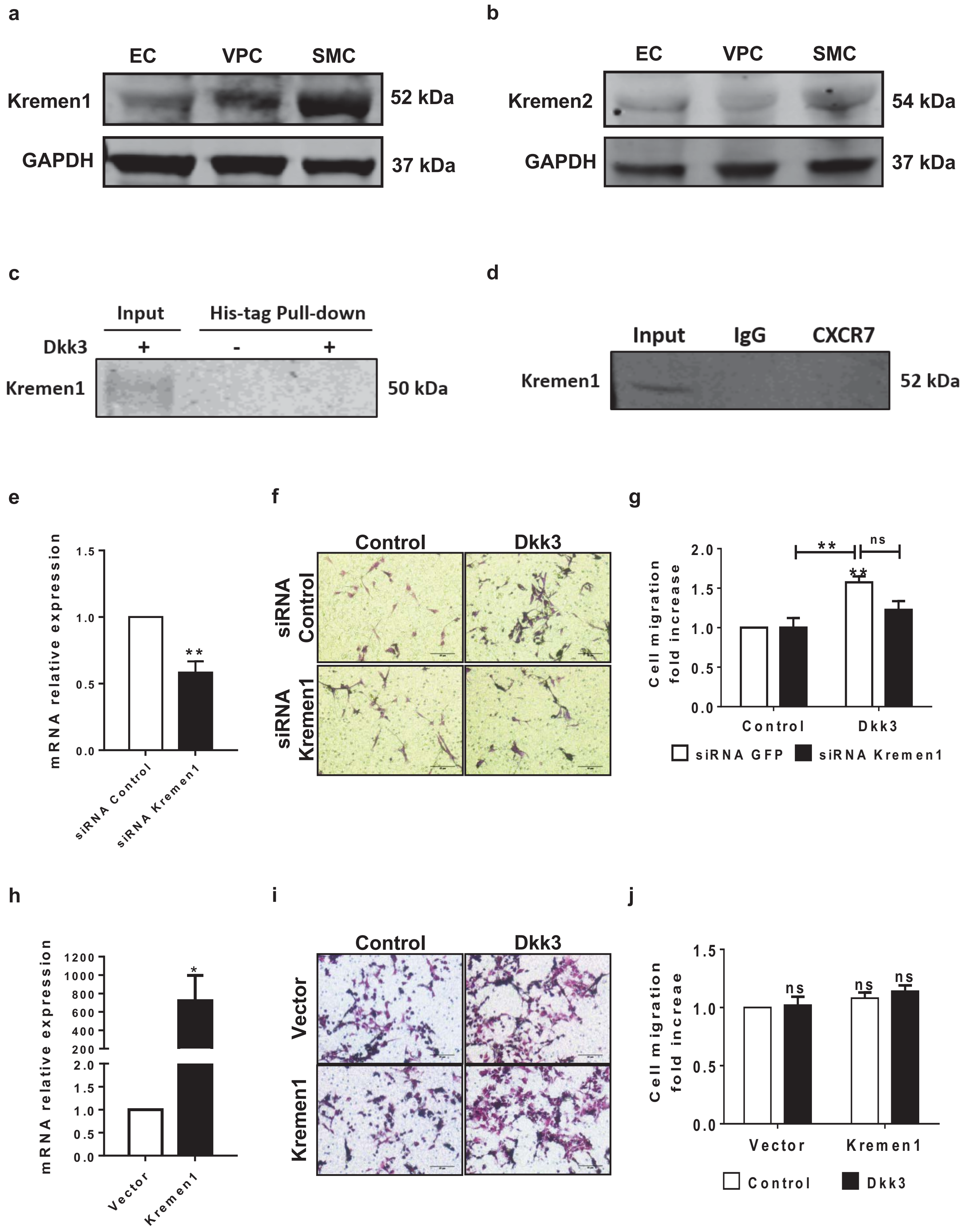
a
EC
VPC
SMC
Kremen1
52 kDa
GAPDH
37 kDa
b
EC
VPC
SMC
Kremen2
54 kDa
GAPDH
37 kDa
c
Input
His-tag Pull-down
Dkk3
+
-
+
Kremen1
50 kDa
d
Input
IgG
CXCR7
Kremen1
52 kDa
e
mRNA relative expression
1.5
1.0
0.5
0.0
**
siRNA Control
siRNA Kremen1
f
Control
Dkk3
siRNA Control
siRNA Kremen1
g
Cell migration fold increase
2.0
1.5
1.0
0.5
0.0
**
ns
**
Control
Dkk3
siRNA GFP
siRNA Kremen1
h
mRNA relative expression
1200
1000
800
600
400
200
2.0
1.5
1.0
0.5
0.0
*
Vector
Kremen1
i
Control
Dkk3
Vector
Kremen1
j
Cell migration fold increae
1.5
1.0
0.5
0.0
ns
ns
ns
Vector
Kremen1
Control
Dkk3

Supplementary Figure IV contimues

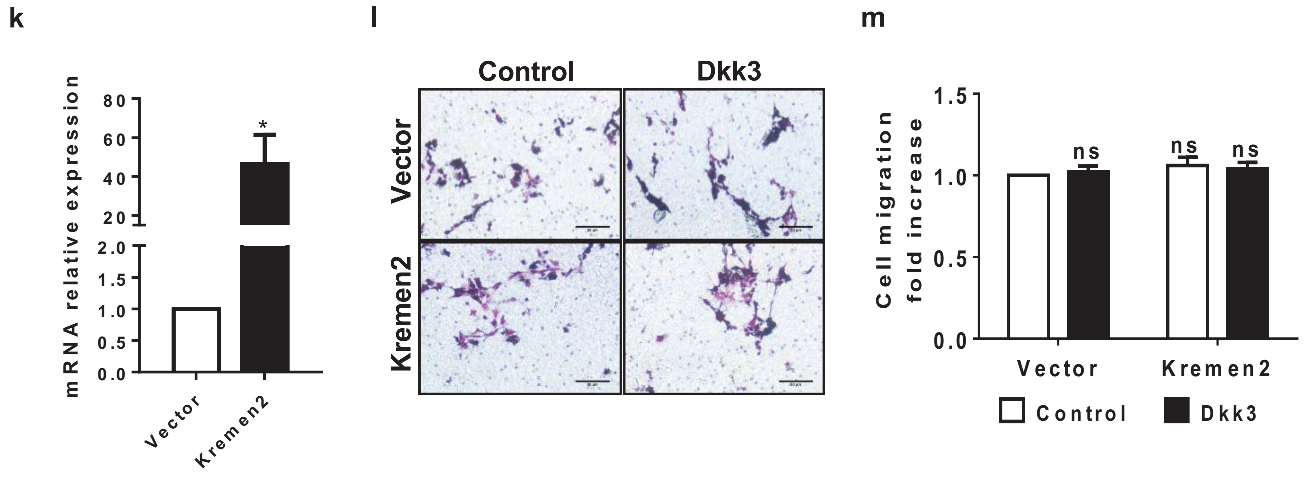

Online figure IV. Kremen1/2 are not involved in Dkk3-mediated migration of Sca-1+ VPCs. (**a, b**) Western blot analysis of Kremen1 and Kremen2 expression in murine endothelial cells (EC), Sca-1+ cells (VPC) and smooth muscle cells (SMC). Kremen1 is expressed in Sca-1+ cells, whereas Kremen2 expression is low. (**c**) Immunoblotting of Kremen1 pulled-down from Dkk3-His tagged, using Nickel magnetic beads. Sca-1+ cells were treated with Dkk3-His tagged. Kremen1 is not pulled-down with Dkk3-His-tagged. (**d**) Co-Immunoprecipitation of Kremen1 with CXCR7. Kremen1 does not co-immunoprecipitate with CXCR7 from Sca-1+ cells. (**e**) Quantitative RT-qPCR analysis of Kremen1 expression in Sca-1+ cells transfected with siRNA targeting Kremen1. Expression levels were normalized against GAPDH (n=5, two-tailed Student's t-test). (**f**) Representative images of transwell migration assay of Sca-1+ cells transfected with Kremen1 siRNA in response to Dkk3 (25 ng/mL). (**g**) Quantitative analysis of the migrated cells in response to Dkk3 upon Kremen1 downregulation (n=4, Two-way ANOVA followed by Bonferroni's test). Kremen1 knockdown does not affect significantly Dkk3-mediated migration of Sca-1+ VPCs. (h, k)) Quantitative RT-qPCR analysis of Kremen1 and Kremen2 mRNA expression in HEK 293T cells transfected with, respectively, Kremen1 and Kremen2 expression plasmids. Expression levels were normalized against GAPDH (n=4, two-tailed unpaired Student's t-test). (i, l) Representative images of transwell migration assay of HEK 293T cells overexpressing Kremen1 and Kremen2, respectively, in response to Dkk3 stimulation. (j, m) Quantitative analysis of the transwell migration assays. Dkk3 is not able to induce migration of Kremen1 or Kremen2 overexpressing HEK 293T cells. (n=5, Two-way ANOVA followed by Bonferroni's post-hoc test). The data are expressed as the mean±s.e.m. of 4 to 5 independent experiments. *P<0.05, **P<0.01, compared with control group.

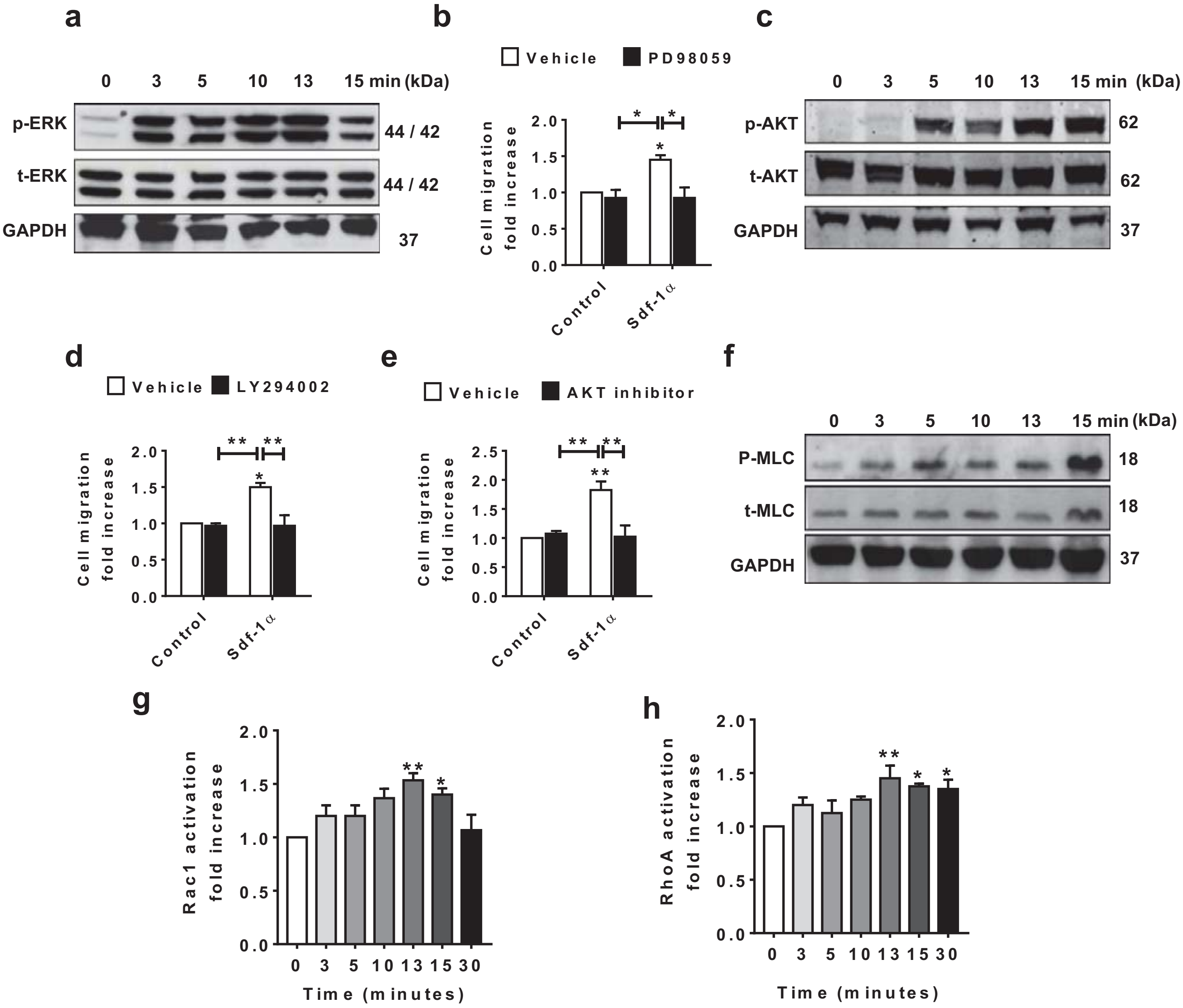

Online Figure V. Dkk3 and Sdf-1α activate similar signalling pathways. (**a**) Western blot analysis of phosphorylated and total ERK1/2 proteins in Sca-1+ cells stimulated with Sdf-1α at the indicated time points. ERK1/2 phosphorylation level increases in response to Sdf-1α stimulation. (**c**) Western blot analysis of phosphorylated and total AKT proteins in Sca-1+ cells treated with Sdf-1α at the time points indicated. AKT phosphorylation is induced with Sdf-1α treatment. (**b**, **d, e**) Quantification of Sdf-1α-driven Sca-1+ cell migration upon treatment with, respectively, PD98059 (10 μM, n=4), LY294002 (10 μM, n=3) and AKT inhibitor (2.5 μM, n=4). Sdf-1α-driven cell migration is abrogated when the cells are treated with the inhibitors. (**f**) Western blot analysis of MLC phosphorylation level in response to Sdf-1α stimulation. MLC phosphorylation is induced by Sdf-1α at the time points indicated. (**g**, **h**) Quantification of, respectively, Rac1-GTP (n=3) and RhoA-GTP (n=4) levels in Sca-1+ cells treated with Sdf-1α at the time points indicated. Sdf-1α promotes Rac1 and RhoA activation, particularly at 13-15 minutes of stimulation. The western blot images are representative of 3 independent experiments. The data are expressed as the mean±s.e.m. of 3 to 4 independent experiments. *P<0.05, **P<0.01, compared with control group (0 ng/mL) (Two-way ANOVA for **b**, **d** and **e** and One-way ANOVA for **g** and **h**, followed by Bonferroni's post-hoc test).

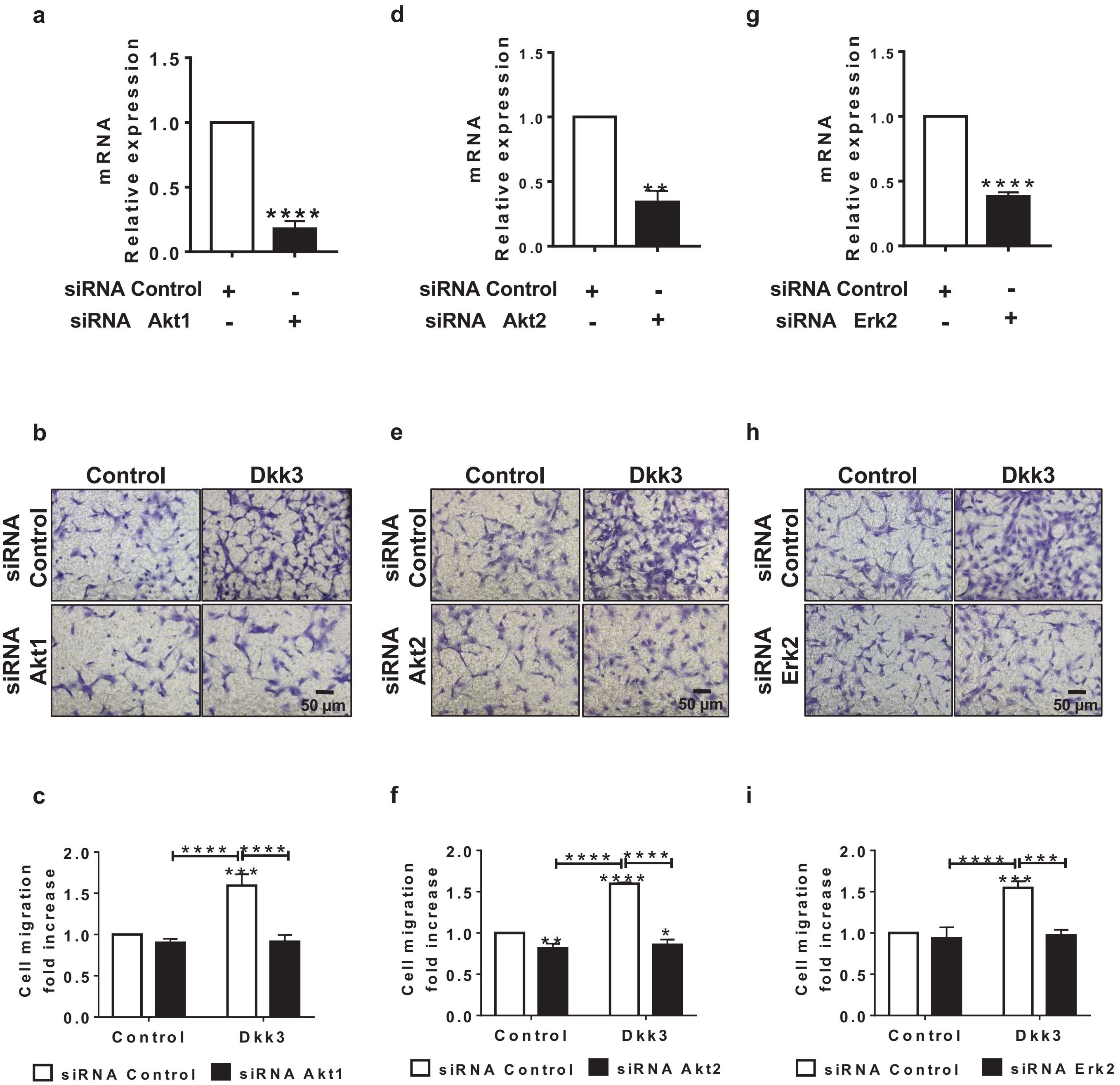

Online Figure VI. AKT and ERK signaling pathways are involved in Dkk3-driven migration of Sca-1+ VPCs. (**a, d, g**) Quantitative RT-qPCR analysis of Akt1, Akt2 and Erk2 expression in Sca-1+ cells transfected with siRNA targeting Akt1, Akt2 and Erk2, respectively. Expression levels were normalized against GAPDH (n=3, two-tailed Student's t-test). (**b**, **e**, **h**) Representative images of transwell migration assay of Sca-1+ cells transfected with Akt1, Akt2 and Erk2 siRNA, respectively, in response to Dkk3 (25 ng/mL) treatment. (**c, f, i**) Quantitative analysis of the migrated cells in response to Dkk3 treatment (n=3, Two-way ANOVA followed by Bonferroni's test). The data are expressed as the mean±s.e.m. of 3 independent experiments. *P<0.05, **P<0.01, ***P<0.001, ****P<0.0001, compared with control group (0 ng/mL).

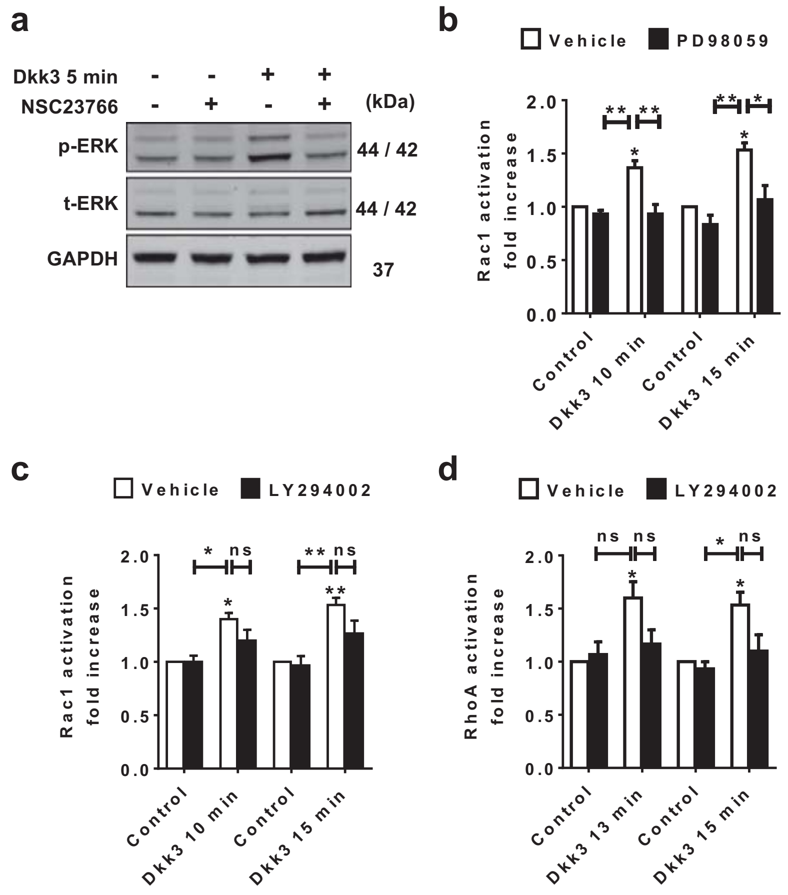

Online Figure VII. Feedback mechanism between ERK1/2 and Rho GTPases, but not between PI3K/AKT and Rho GTPases, in Dkk3-mediated migration of Sca-1+ cells. (**a**) Western blot analysis of Dkk3-mediated ERK1/2 phosphorylation upon NSC23766 treatment, at the time points indicated. Rac1 activation inhibitor NSC23766 supresses ERK1/2 phosphorylation induced by Dkk3. Western blot images are representative of 3 independent experiments. (**b**) Quantification of Rac1 activation triggered by Dkk3 in response to PD98059 treatment. MEK/ERK1/2 inhibitor PD98059 represses Rac1-GTP level increase induced by Dkk3, at the time points considered (n=3). (**c**, **d**) Quantification of Dkk3-triggered activation of Rac1 and RhoA, respectively, in Sca-1+ cells, upon treatment with LY294002 (10 μM, n=3). PI3K/AKT inhibition with LY294002 does not affect the activation of Rho GTPases induced by Dkk3. The data are expressed as the mean±s.e.m. of 3 independent experiments. *$P<0.05$, **$P<0.01$, compared with control group (0 ng/mL) (Two-way ANOVA, followed by Bonferroni's post-hoc test).

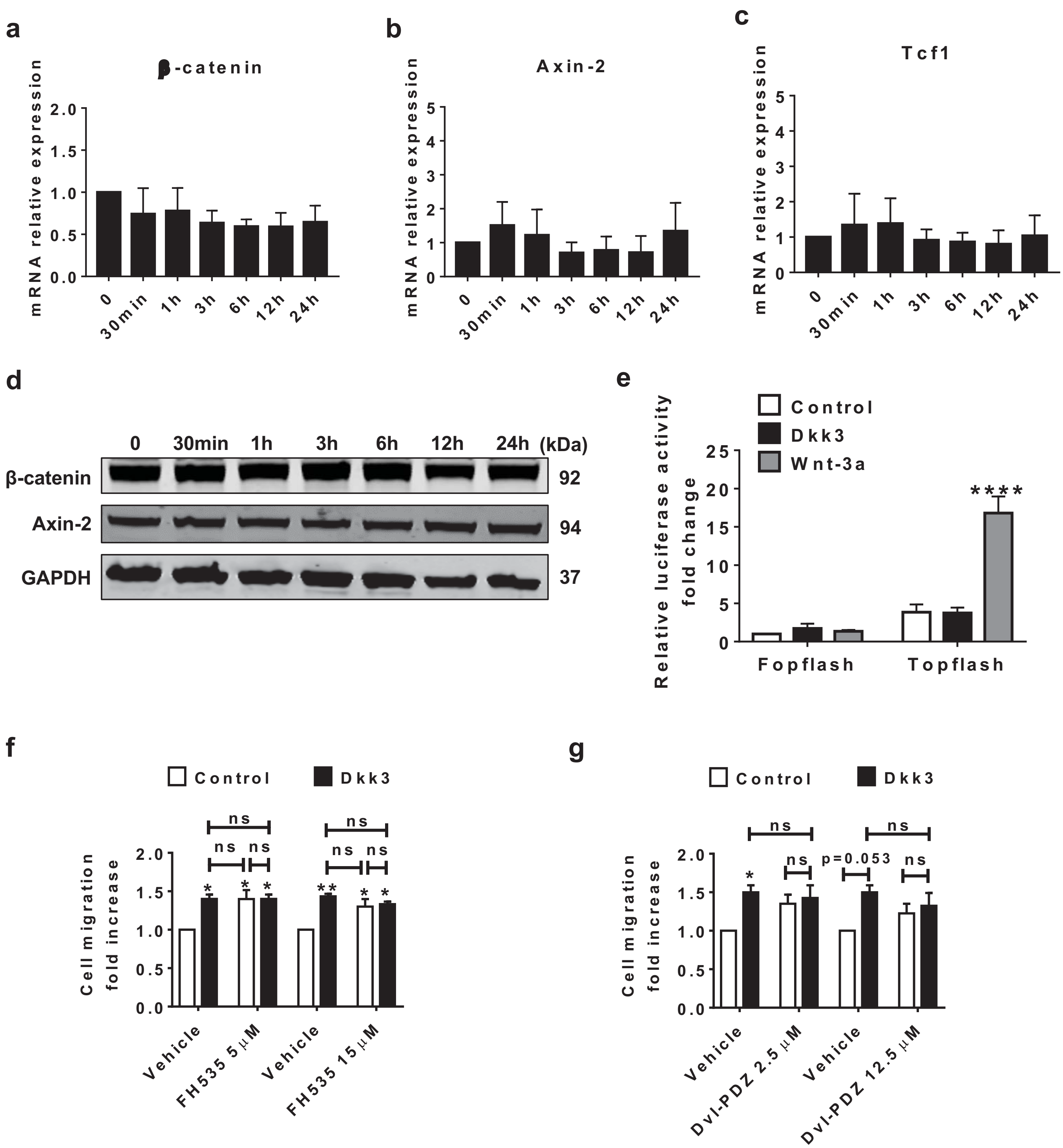

Online Figure VIII. The Wnt signalling pathway is not involved in Dkk3-driven migration of Sca-1+ VPCs.(**a**, **b**, **c**) Quantitative RT-qPCR analysis of β-catenin, Axin-2 and Tcf1 expression in Sca-1+ cells stimulated with Dkk3 (25ng/ml) at the indicated time points. Expression levels were normalized against GAPDH (n=3, One-way ANOVA followed by Bonferroni's multiple comparisons test), compared with control group (0 ng/mL). (**d**) Western blot analysis of β-catenin and Axin-2 proteins in Sca-1+ cells stimulated with Dkk3 (25ng/mL) at the indicated time points. (**e**). Topflash/Fopflash assay with Dkk3 (25 ng/mL) treatment for 24 hours. Wnt3a (100ng/mL) was used as the positive control. Unlike Wnt3a, Dkk3 did not induce the relative luciferase activity, indicating that Dkk3 does not activate the transcription of Wnt target genes (n=6, Two-way ANOVA followed by Bonferroni's test). (**f**) Quantification of Dkk3-driven Sca-1+ cell migration upon treatment with FH575 (5 μM, n=3). Inhibition of the Wnt/β-Catenin signalling does not affect Dkk3-driven migration rate of Sca-1+ cells. (**g**) Quantification of Dkk3-driven Sca-1+ cell migration in response to treatment with the inhibitor Dvl-PDZ (5μM, n=4). Inhibition of the PDZ domain of the Dishevelled protein involved in both canonical and non-canonical Wnt signalling pathways does not affect Dkk3-driven cell migration of Sca-1+ cells (Two-way ANOVA, followed by Bonferroni's post-hoc test). The data are expressed as the mean±s.e.m. of 3-6 independent experiments. *P<0.05, **P<0.01, ****P<0.0001, compared with control group (0 ng/mL).

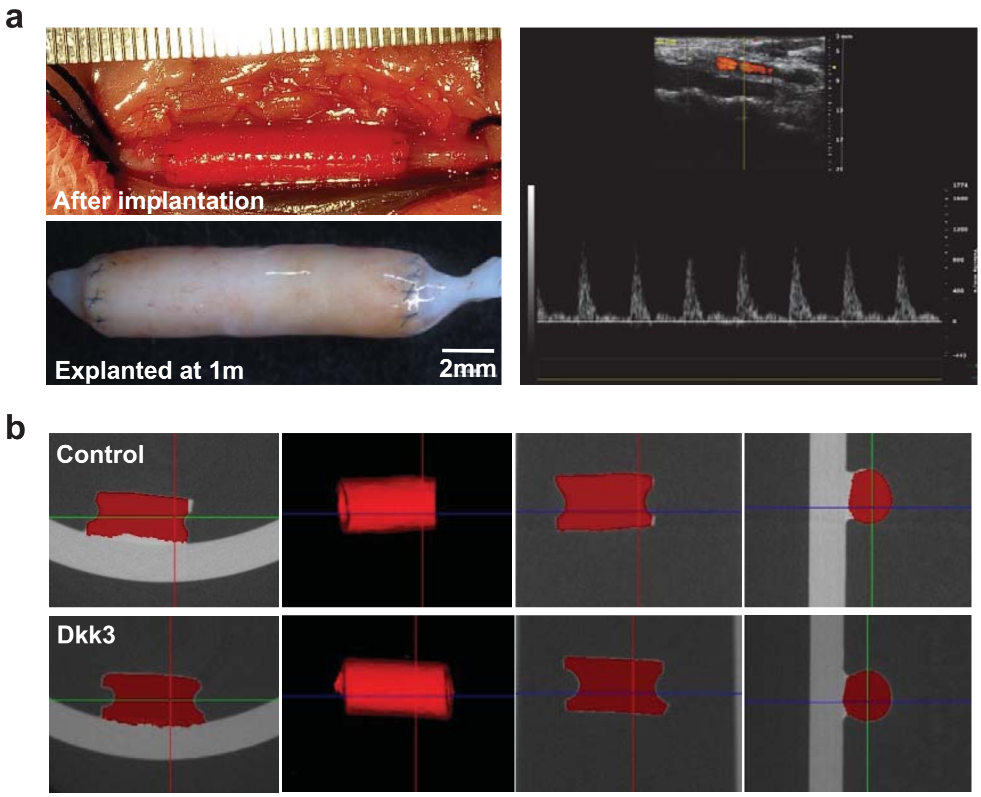

c

Patency rate of implanted tissue-engineered vascular grafts

	2w	1m	3m
Control	4/4	4/4	4/5
Dkk3	4/4	4/5	5/5

Online Figure IX. Characterization and patency of Dkk3-loaded TEVGs in rat model. (**a**) Images of Dkk3-loaded vascular grafts (1 cm in length) implanted to replace a segment of the abdominal aorta in rat (top left), explanted after 1 month (bottom left) and of ultrasound scanning of the implanted grafts (right). (**b**) Micro-CT images of the level of calcification of the grafts at 3 months. Calcification is not detected in both Control and Dkk3 groups. (**c**) Determination of the patency rate of the implanted TEVGs. The patency rate at different time points is similar between both groups, with no aneurysm or occlusion detected.

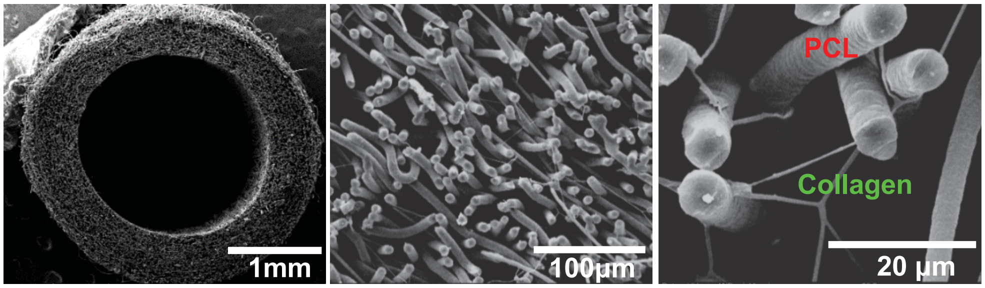

Online Figure X. The micro-structure of tissue engineered vessel grafts (TEVGs). Scanning electronic microscopy images of the hybrid fibrous structure of the TEVGs. The TEVGs consist of PCL microfibers and collagen nanofibers.

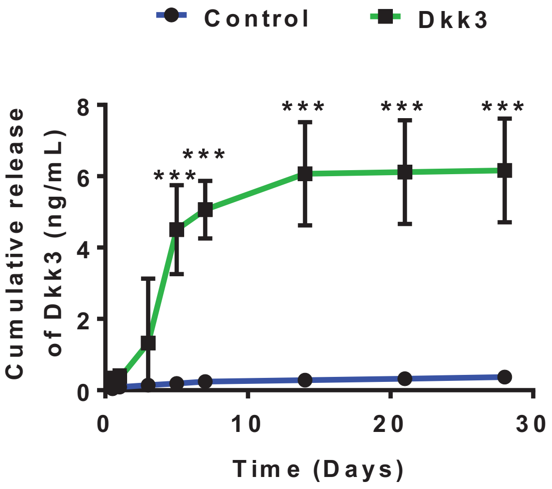

Online Figure XI. Dkk3 is released in a sustained manner from the TEVGs. Measurement of Dkk3 release *in vitro*. Dkk3 is released over time in a sustained manner from the TEVGs. The data are expressed as the mean±s.e.m. of 3 independent experiments. ***P<0.001, compared with control group (0 ng/mL) (Two-way ANOVA repeated measures, followed by Bonferroni's post-hoc test).

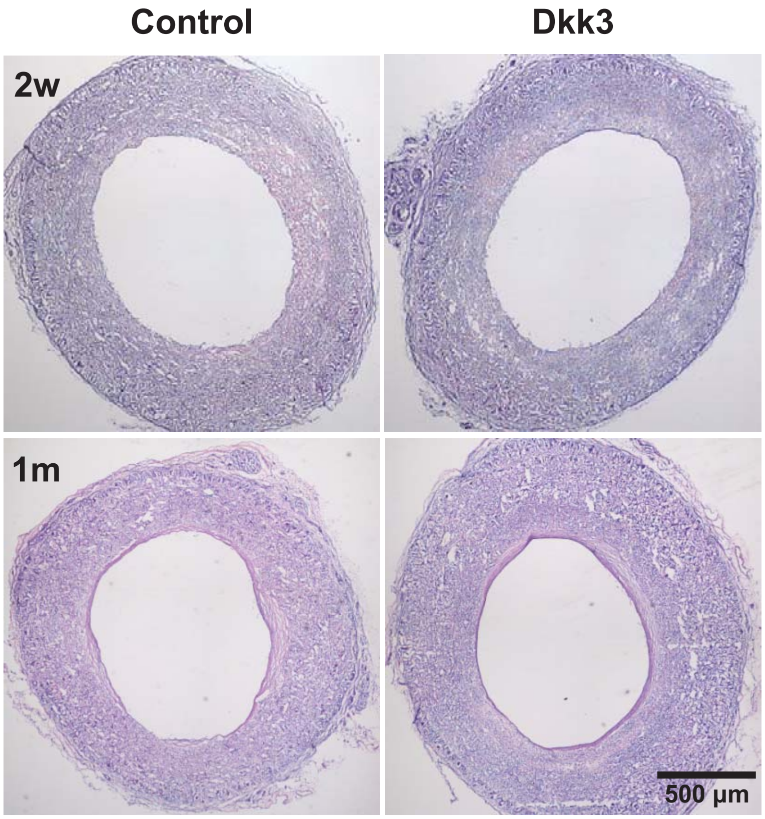

Online Figure XII. Dkk3 delivery by the TEVGs does not lead to intimal hyperplasia. H&E staining on the cross-sections of Control- and Dkk3-TEVGs at 2 and 4 weeks post-implantations. No evident intimal hyperplasia is observable in Dkk3-TEVGs compared to control group.

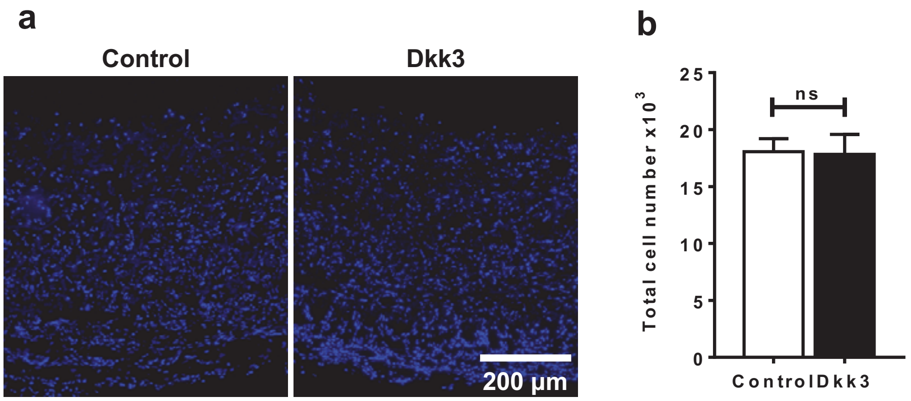

Online Figure XIII. Cell infiltration into TEVGs after 2 weeks post implantation.

(**a**) Representative immunofluorescence images showing cell infiltration into the TEVG at 2 weeks analyzed by DAPI staining of the cross-sections. (b) Quantification of cell number in the TEVGs. No difference is found between Control and Dkk3 groups. The data are expressed as the mean±s.e.m. of 4 independent experiments (4 grafts for each group) (Two-tailed unpaired Student's t-test).

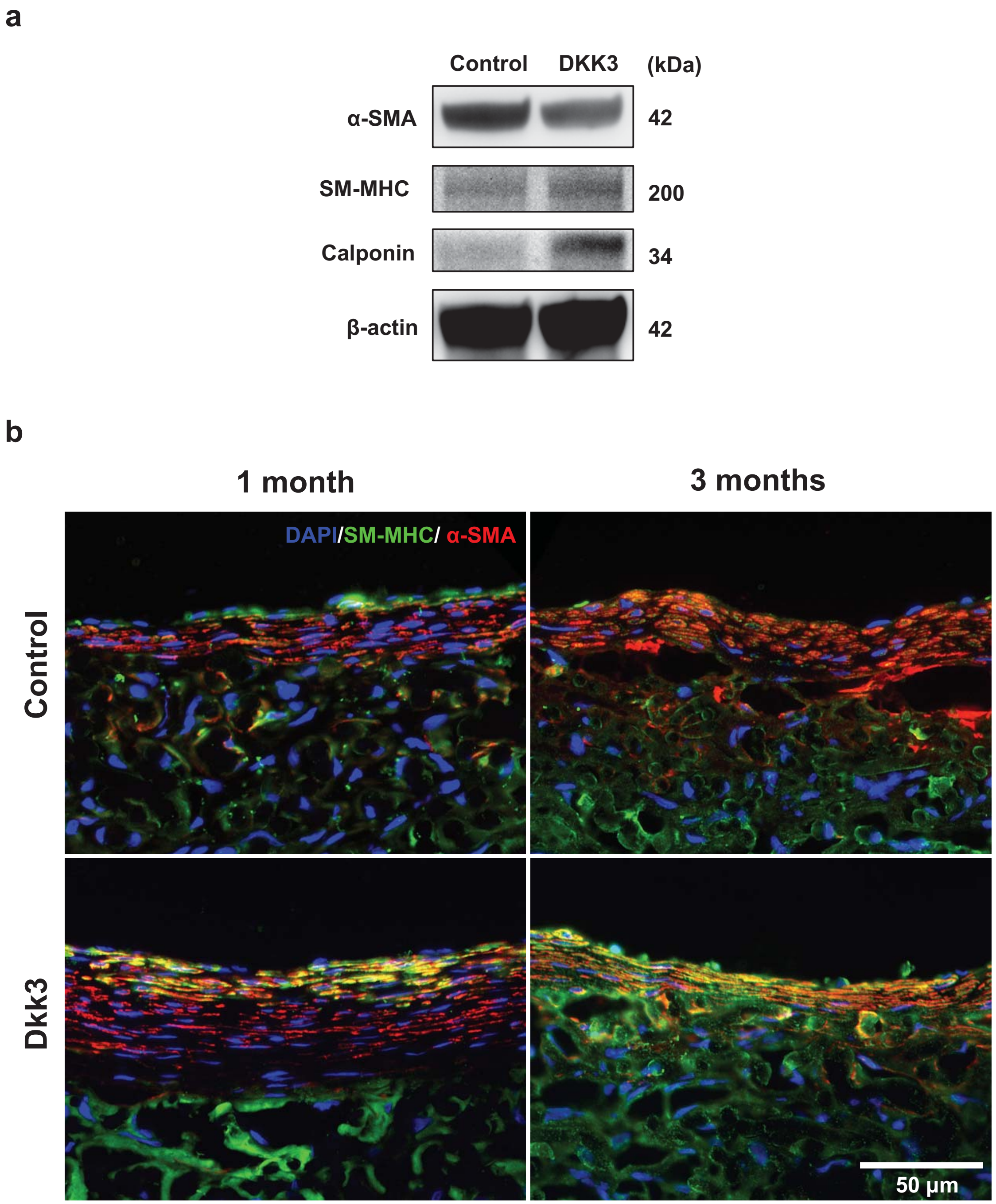

Online Figure XIV. Dkk3 promotes the transition of SMCs towards a more mature and contractile phenotype. (**a**) Western blot analysis of α-SMA, SM-MHC and Calponin expression in explanted control- and Dkk3-TEVGs after 3-months post-implantation. Mature SMCs markers are highly expressed in Dkk3-TEVGs in comparison with Control-TEVGs. (**b**) Representative immunofluorescence images of the cross-sections of the TEVGs at 1- and 3-months post-implantation, analyzed by staining with anti-α-SMA (red) and anti-SMMHC (green) antibodies. DAPI was included to counterstain the nuclei. Dkk3-loaded TEVGs display an increased staining of double positive cells (α-SMA+/SM-MHC+), particularly at 3 months post-implantation, in comparison with the control group, thus revealing the regeneration of a more contractile smooth muscle in the TEVGs.

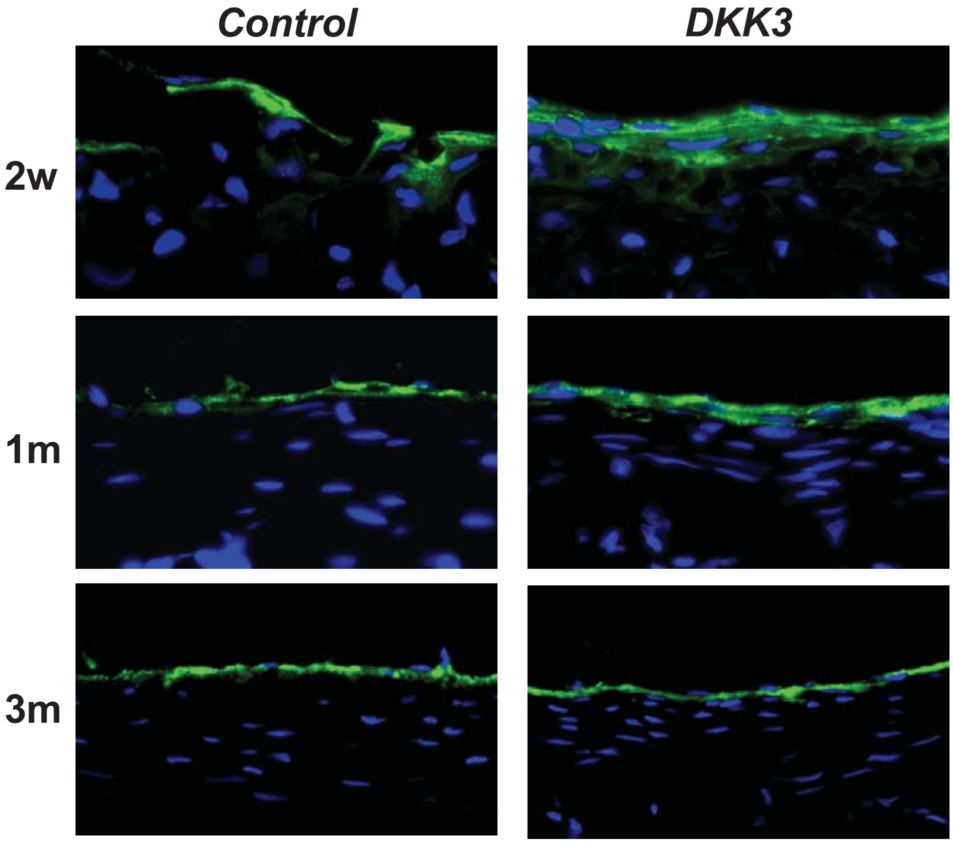

Online Figure XV. Endothelization of the Dkk3-loaded TEVGs. Representative immunofluorescence images of the TEVGs at 2weeks, 1 and 3 months post-implantation with staining against vWF (green). DAPI was included to counterstain the nuclei. A more uniform and continuous endothelization is observed in the lumen of Dkk3-TEVGs, in comparison to control group.

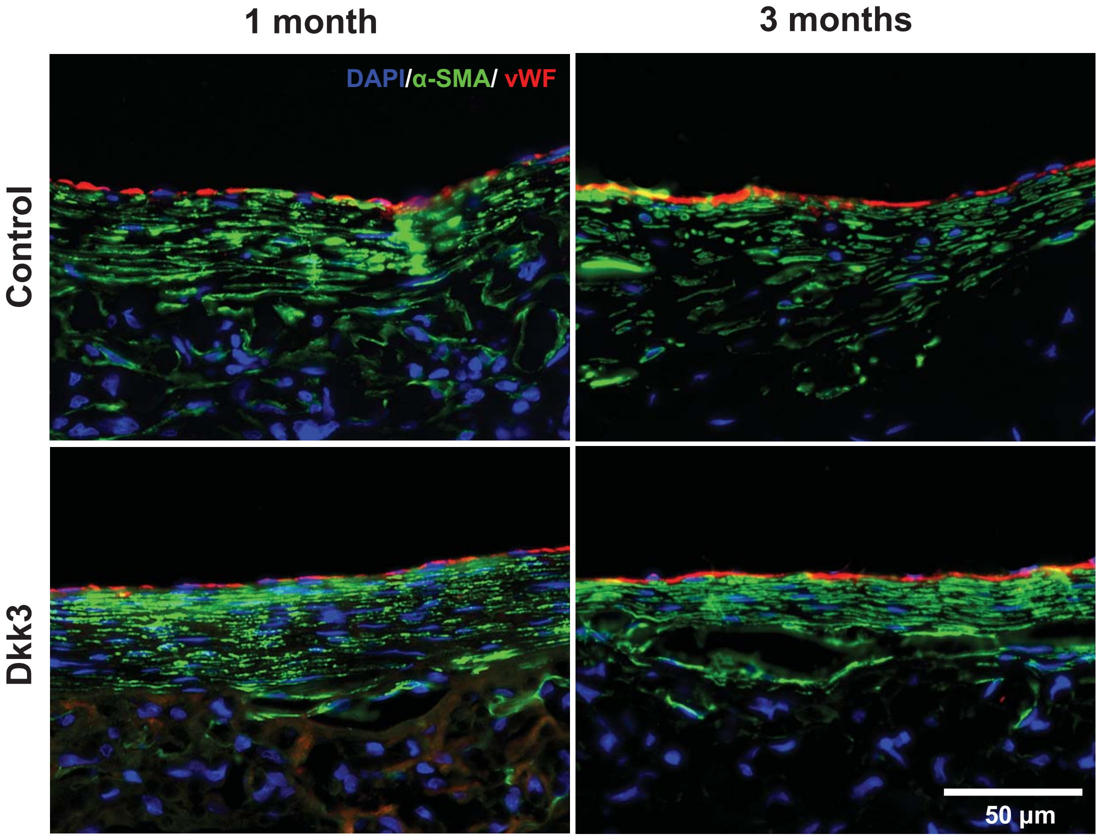

Online Figure XVI. Dkk3 increases EC-SMC interaction in the TEVGs. Representative immunofluorescence images of the cross-section of the TEVGs, at 1 and 3 months post-implantation, analyzed by staining with anti-α-SMA (green) and anti-vWF (red) antibodies. DAPI was included to counterstain the nuclei. In Dkk3-TEVGs the SMC layers are better aligned beneath the endothelium and reveal a better interaction with the EC layer, in comparison with the control group.

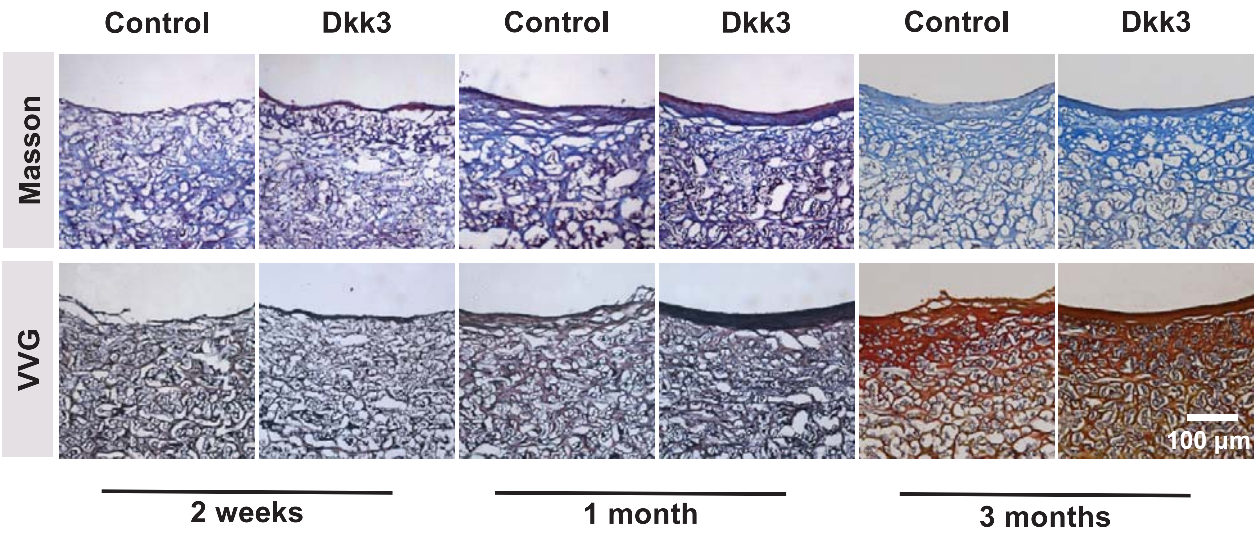

Online Figure XVII. Dkk3 increases the secretion of extracellular matrix in the TEVGs. Masson's trichrome (upper) and VVG (below) staining on cross-sections of the TEVGs. The deposition of collagen and elastin in the TEVGs, at the time points indicated, is apparently greater in Dkk3-TEVGs than in Control-TEVGs.

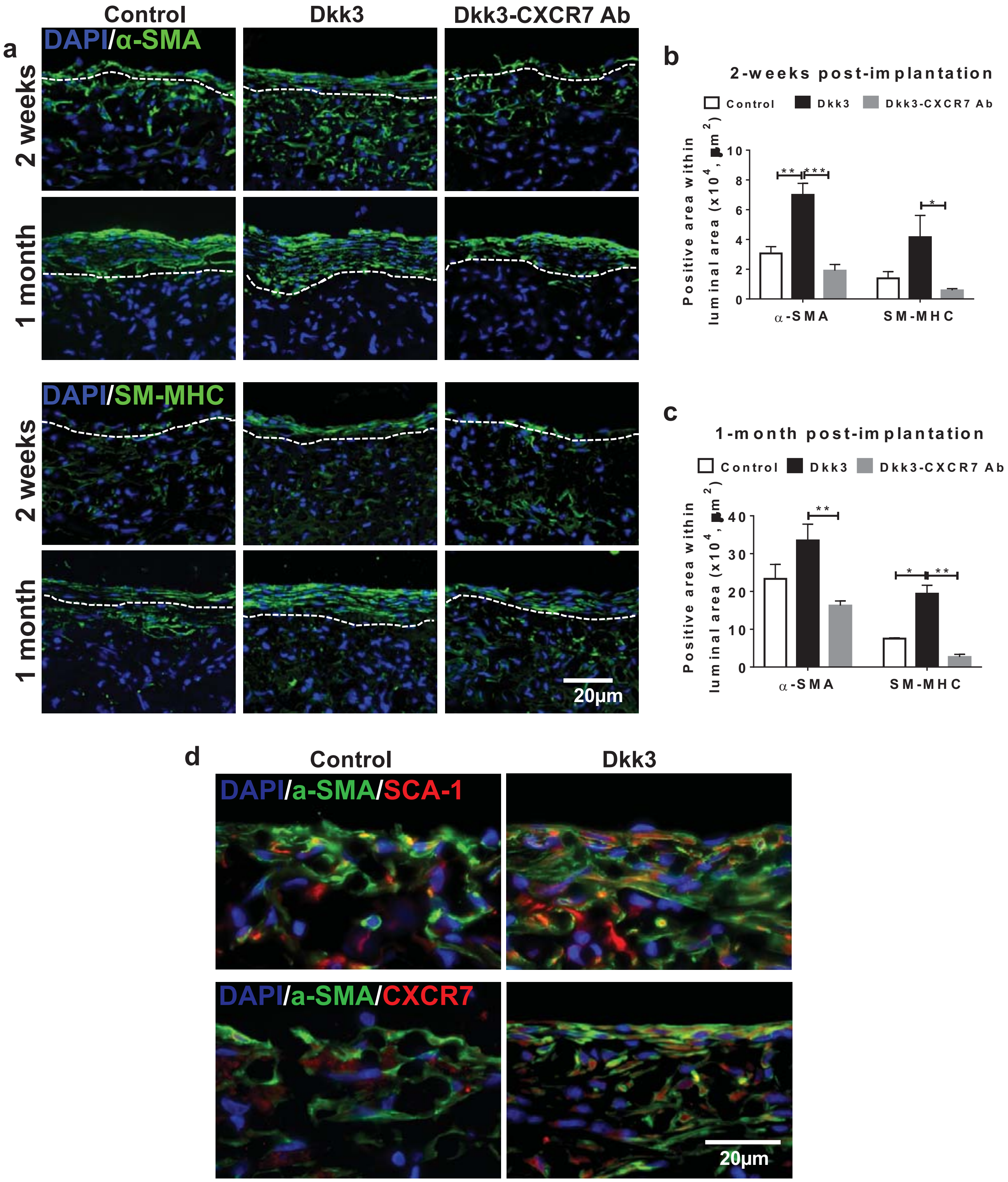

Online Figure XVIII. Dkk3 enhances smooth muscle regeneration with CXCR7 involvement. (**a**) Representative immunofluorescence images of control-, Dkk3- and Dkk3-CXCR7Ab-TEVGs showing SMC recruitment by staining the sections with anti-α-SMA and anti-SMMHC antibodies at 2weeks and 1 month. DAPI was used to counterstain the nuclei. (**b**, **c**) Quantification of smooth muscle regeneration by determination of the total area of α-SMA and of SM-MHC positive cells in the luminal region (delineated by the white dashed lines) at different time points. The images are representative of 4 grafts for each group. The data are expressed as the mean±s.e.m. of 4 samples. *P<0.05, **P<0.01, ***P<0.001 (Two-way ANOVA followed by Bonferroni's test). **(d)** Representative immunofluorescence images of the cross-sections of TEVGs exhibiting double immunostaining with anti-α-SMA (green) and anti-Sca-1 (red) antibodies, anti-α-SMA (green) and anti-CXCR7 (red) antibodies at 2 weeks.

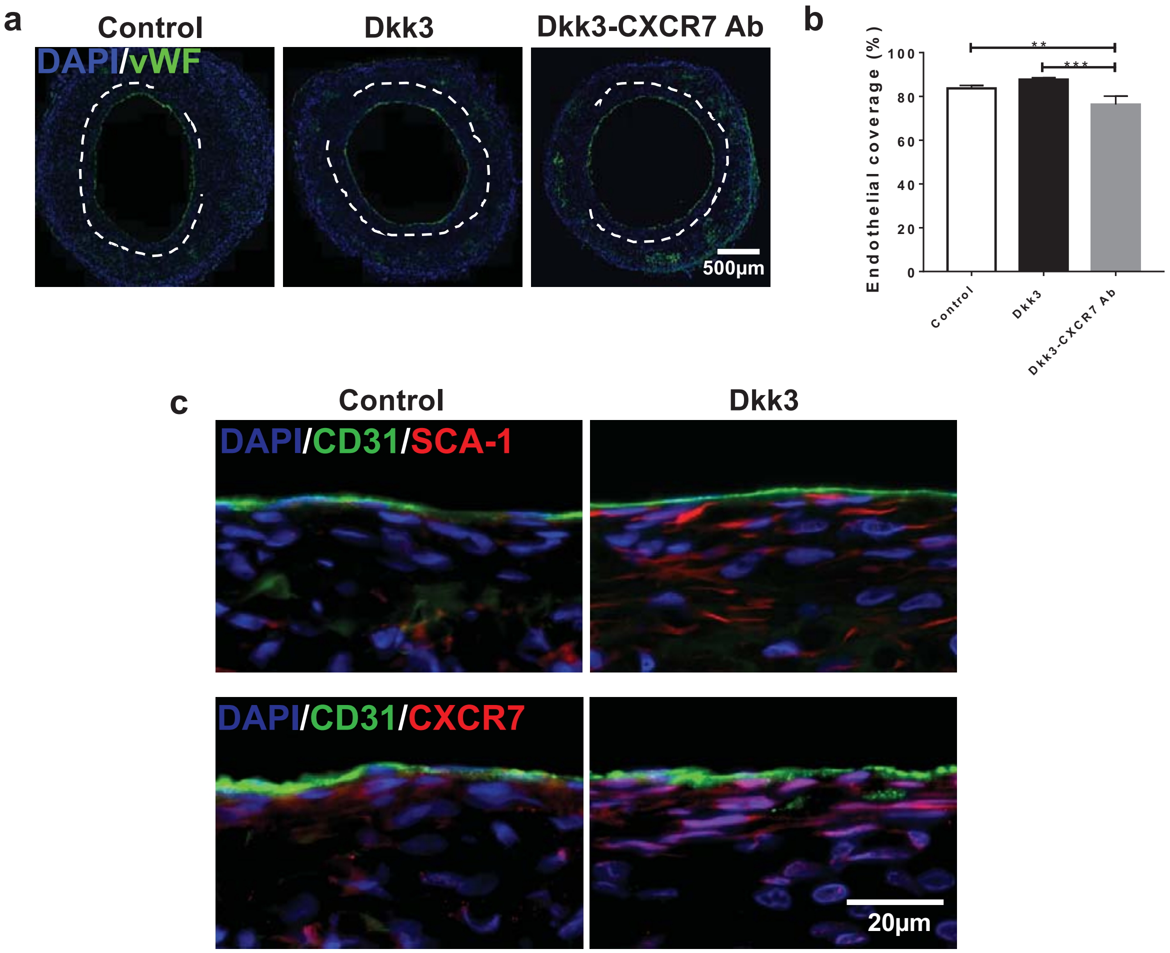

Online Figure XIX. CXCR7 is involved in the endothelialization of the TEVGs. (**a**) Representative immunofluorescence images of control-, Dkk3- and Dkk3-CXCR7Ab-TEVGs showing endothelial regeneration by staining the sections with anti-vWF antibodies after 1 month implantation. DAPI was used to counterstain the nuclei. White line indicated a thin vWF+ layer which suggest to be mature and real endothelium. Those thicker layer composed of diffused vWF+ cells were macrophage endocytosing platelets, can not identified as true endothelium. (**b**) Quantification of endothelial coverage by determine the percentage vWF positive cells of total length. The images are representative of 4 grafts for each group. The data are expressed as the mean±s.e.m. of 4 samples. **P<0.01, ***P<0.001 (One-way ANOVA followed by Turkey's multiple comparisons test). **(c)** Representative immunofluorescence images of the sections of TEVGs exhibiting double immunostaining with anti-CD31 (green) and anti-Sca-1 (red) antibodies, anti-CD31 (green) and anti-CXCR7 (red) antibodies at 1month.

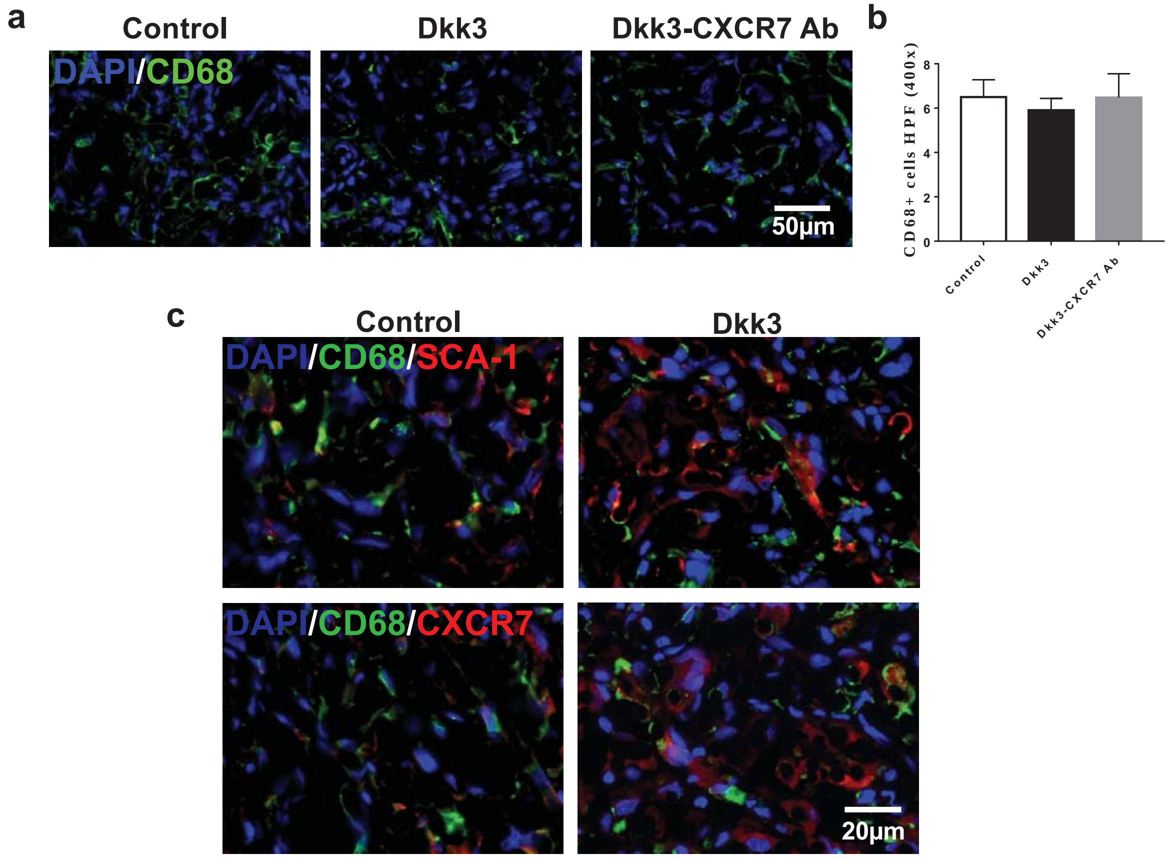

Online Figure XX. Dkk3 has no effect on macrophage infiltration. (**a**) Representative immunofluorescence images of control-, Dkk3- and Dkk3-CXCR7Ab-TEVGs showing macrophage recruitment by staining the sections with anti-CD68 antibodies after 1 month implantation. DAPI was used to counterstain the nuclei. (**b**) Quantification of macrophage infiltration into the three types of TEVGs. The images are representative of 4 grafts for each group. The data are expressed as the mean±s.e.m. of 4 samples. (One-way ANOVA followed by Kruskal-Wallis multiple comparisons test). **(c)** Representative immunofluorescence images of the cross-sections of TEVGs exhibiting double immunostaining with anti-CD68 (green) and anti-Sca-1 (red) antibodies, anti-CD68 (green) and anti-CXCR7 (red) antibodies at 1 month.

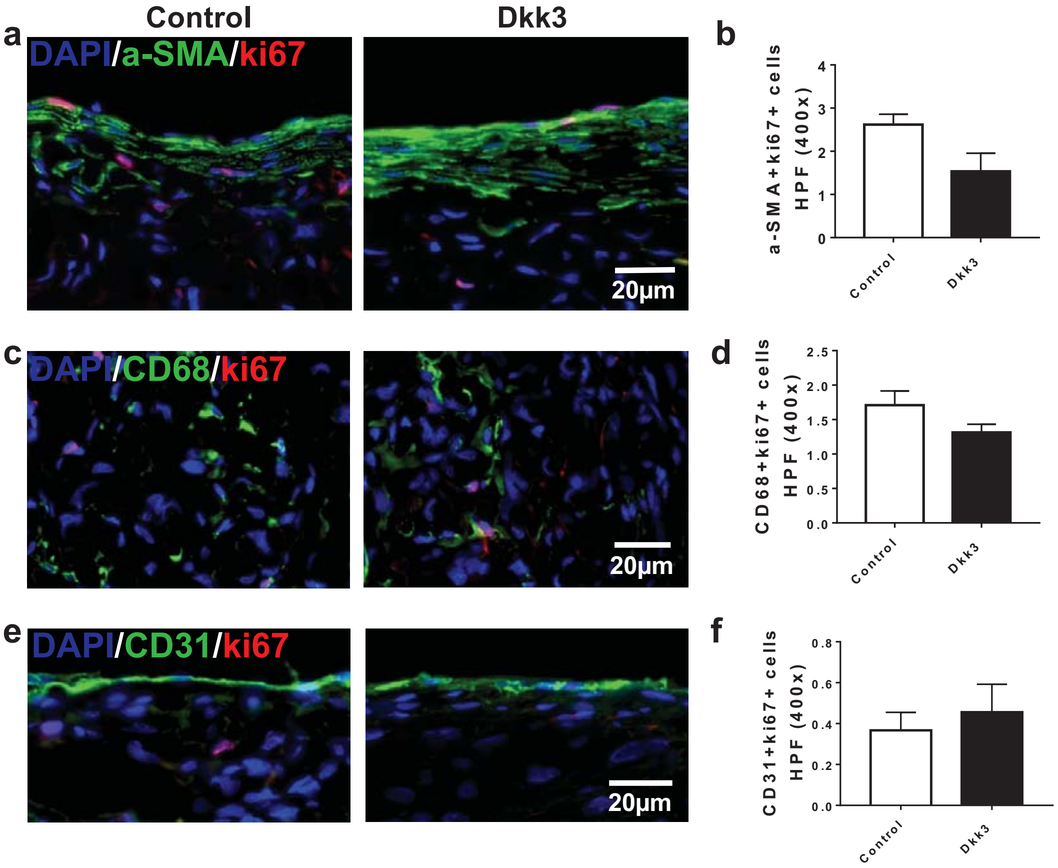

Online Figure XXI. Dkk3 has no effect on the proliferation of different cell types. (**a, c, e**) Representative immunofluorescence images of the cross-sections of TEVGs exhibiting double immunostaining with anti-α-SMA (green) and anti-ki67 (red) antibodies, anti-CD68 (green) and anti-ki67 (red) antibodies, anti-CD31 (green) and anti-ki67 (red) antibodies after 1 month implantation. DAPI was used to counterstain the nuclei. (**b, d, f**) Quantification of proliferate SMCs, macrophage and ECs into the two types of TEVGs. The images are representative of 4 grafts for each group. The data are expressed as the mean±s.e.m. of 4 samples (Mann Whitney test).

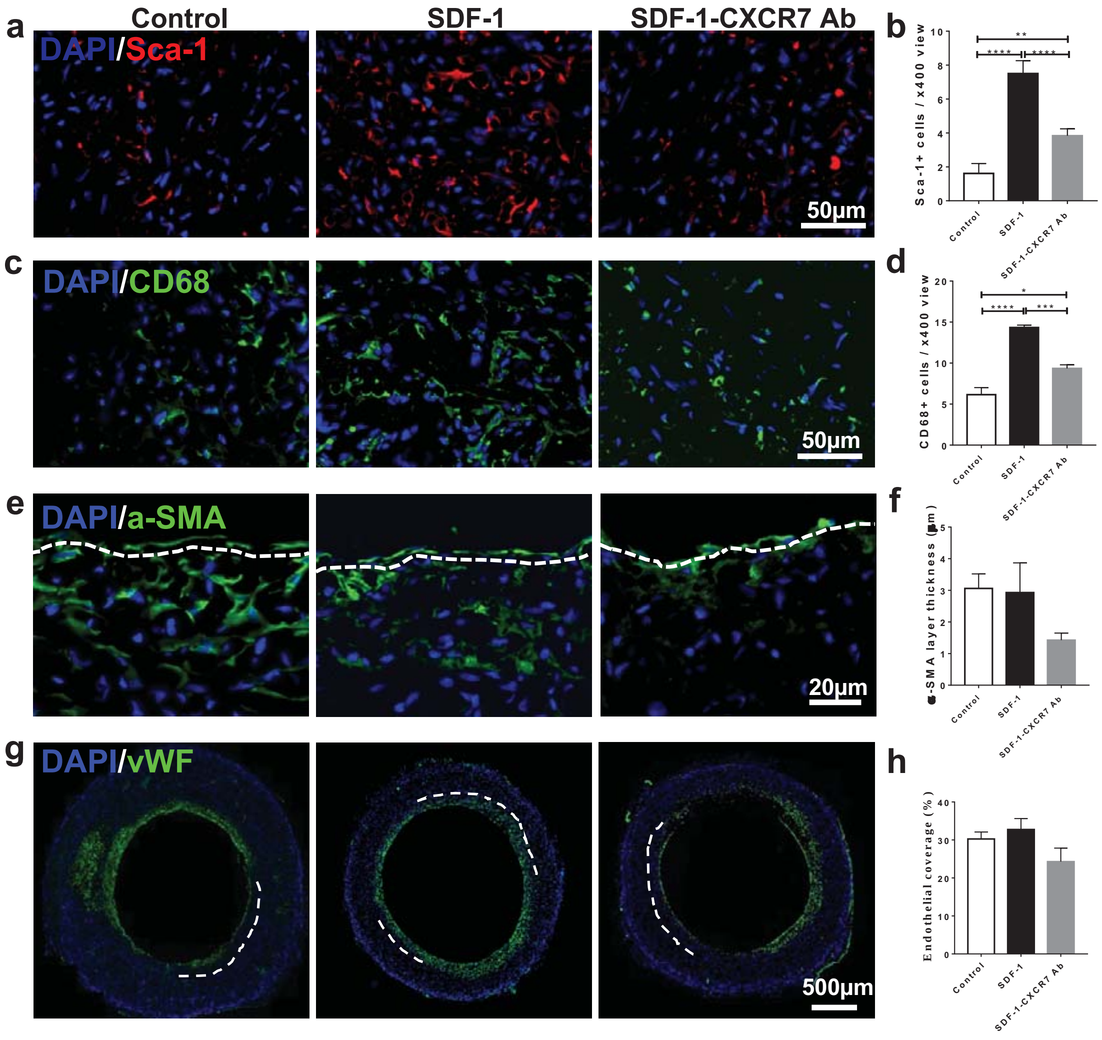

Online Figure XXII. Sdf-1α promotes Sca-1+ cells recruitment into the TEVGs at 2 weeks post-implantation. (**a**) Representative immunofluorescence images of Control, SDF-1- and SDF-1-CXCR7 Ab TEVGs showing Sca-1 progenitor cells recruitment by staining the sections with anti-sca-1 antibodies. DAPI was used to counterstain the nuclei. **(b)** Quantification of Sca-1+ progenitor cell infiltration into the three types of TEVGs. (**c**) Representative immunofluorescence images of TEVGs showing macrophage recruitment by staining the sections with anti-CD68 antibodies. **(d)** Quantification of macrophage infiltration into the three types of TEVGs. (**e**) Representative immunofluorescence images of TEVGs showing SMC recruitment by staining the sections with anti-α-SMA antibodies. (**f**) Quantification of smooth muscle regeneration by determination of the total area of α-SMA positive cells in the luminal region (delineated by the white dashed lines). (**g**) Representative immunofluorescence images of TEVGs showing endothelial regeneration by staining the sections with anti-vWF antibodies. (**h**) Quantification of endothelial coverage by determine the percentage vWF positive cells of total length. The images are representative of 4 grafts for each group after 2 weeks implantation. The data are expressed as the mean±s.e.m. of 4 samples. *P<0.05, **P<0.01, ***P<0.001, ****P<0.0001 (One-way ANOVA followed by Turkey's multiple comparisons test for b, d, f and h).
